# Caspase cleavage of RIPK3 after Asp^333^ is dispensable for mouse embryogenesis

**DOI:** 10.1038/s41418-023-01255-5

**Published:** 2024-01-08

**Authors:** Kim Newton, Katherine E. Wickliffe, Allie Maltzman, Debra L. Dugger, Joshua D. Webster, Hongyan Guo, Vishva M. Dixit

**Affiliations:** 1 Department of Physiological Chemistry, Genentech, 1 DNA Way, South San Francisco, CA 94080 USA; 2 Department of Pathology, Genentech, 1 DNA Way, South San Francisco, CA 94080 USA; 3 Department of Microbiology and Immunology, LSU Health Shreveport, Shreveport, LA 71103 USA

**Keywords:** Development, Kinases

## Abstract

The proteolytic activity of caspase-8 suppresses lethal RIPK1-, RIPK3- and MLKL-dependent necroptosis during mouse embryogenesis. Caspase-8 is reported to cleave RIPK3 in addition to the RIPK3-interacting kinase RIPK1, but whether cleavage of RIPK3 is crucial for necroptosis suppression is unclear. Here we show that caspase-8-driven cleavage of endogenous mouse RIPK3 after Asp^333^ is dependent on downstream caspase-3. Consistent with RIPK3 cleavage being a consequence of apoptosis rather than a critical brake on necroptosis, *Ripk3*^*D333A/D333A*^ knock-in mice lacking the Asp^333^ cleavage site are viable and develop normally. Moreover, in contrast to mice lacking caspase-8 in their intestinal epithelial cells, *Ripk3*^*D333A/D333A*^ mice do not exhibit increased sensitivity to high dose tumor necrosis factor (TNF). *Ripk3*^*D333A/D333A*^ macrophages died at the same rate as wild-type (WT) macrophages in response to TNF plus cycloheximide, TNF plus emricasan, or infection with murine cytomegalovirus (MCMV) lacking M36 and M45 to inhibit caspase-8 and RIPK3 activation, respectively. We conclude that caspase cleavage of RIPK3 is dispensable for mouse development, and that cleavage of caspase-8 substrates, including RIPK1, is sufficient to prevent necroptosis.

## Introduction

Apoptosis, pyroptosis, and necroptosis are cell death programs that eliminate infected, damaged, or obsolete cells from multicellular organisms. Toll-like receptor (TLR) 3, TNF receptor 1 (TNFR1), FAS, TRAIL receptors, and the intracellular Z-form nucleic acid sensor ZBP1 can each induce apoptosis by activating the protease caspase-8 [1–7]. In some settings, however, activation of caspase-8 promotes cell survival by suppressing necroptosis signaling. This pro-survival function of caspase-8 is essential for mouse embryogenesis [8–10], antigen receptor-induced proliferation of mouse T cells [9, 11], and proinflammatory gene expression in mouse macrophages exposed to TNF, TLR3 agonist poly(I:C), or TLR4 agonist lipopolysaccharide (LPS) [12, 13]. The catalytic activity of caspase-8 mediates both apoptosis induction and necroptosis suppression [9, 14, 15]. This dual function of caspase-8 appears to safeguard against viruses expressing inhibitors of caspase-8 [16–19]. Viruses seek to preserve their replicative niche by inhibiting caspase-8-dependent host cell death, but in doing so unleash RIPK3- and MLKL-dependent necroptosis.

Death receptors, including TNFR1 and FAS, induce apoptosis by using the adaptor protein FADD to recruit caspase-8, cFLIP, and, in humans, caspase-10 [20, 21]. Dimerization and auto-processing of caspase-8 zymogens yields the fully active protease that cleaves and activates caspase-3 and -7 [22–25]. Many caspase substrates are cleaved and the cell is dismantled into membrane-enveloped apoptotic bodies [26]. Proteolytically active heterodimers of caspase-8 and cFLIP_L_ (the long isoform of cFLIP) [27, 28] are believed to suppress necroptosis by cleaving the kinase RIPK1 [9, 14, 29, 30]. Whether RIPK1 is the only substrate cleaved by caspase-8 to suppress necroptosis is unclear. Cleavage of RIPK1 is thought to perturb interactions between RIPK1 and RIPK3 that would otherwise promote RIPK3 activation, phosphorylation of the pseudokinase MLKL, and cell lysis [31–34].

In addition to suppressing death receptor-induced, RIPK1-dependent activation of RIPK3, caspase-8 also suppresses activation of RIPK3 by TRIF and ZBP1 [35]. TLR3 and TLR4 use the adaptor protein TRIF to engage RIPK3 [12, 13]. ZBP1 is induced by interferons [36] and appears to activate RIPK3 in response to Z-form RNAs produced by certain viruses and endogenous retroviral elements [37–43]. Both TRIF and ZBP1 can activate RIPK3 in the absence of RIPK1 [13, 35, 44], so it is unclear if cleavage of RIPK1 suppresses necroptosis triggered by TRIF or ZBP1. Caspase-8 may cleave and inactivate RIPK3 [45].

Mice lacking either caspase-8 or FADD, or expressing catalytically inactive caspase-8 (C362A or C362S), die around embryonic day 11 (E11) from RIPK1-, RIPK3-, and MLKL-dependent necroptosis [8–10, 14, 15, 44, 46–48]. Mutation of the caspase-8 cleavage site in mouse RIPK1 also causes embryonic lethality [14, 29, 30, 49], consistent with RIPK1 being an important substrate of caspase-8. Here, we show that mutating the caspase cleavage site in mouse RIPK3 does not cause embryonic lethality. Like their wild-type (WT) counterparts, *Ripk3*^*D333A/D333A*^ knock-in macrophages do not undergo necroptosis unless caspases are inhibited. Thus, cleavage of RIPK1, and potentially other caspase-8 substrates upstream of RIPK3, must be sufficient to prevent necroptosis.

## Results

### *Ripk3*^*D333A/D333A*^ mice are viable

**Fig. 1:**
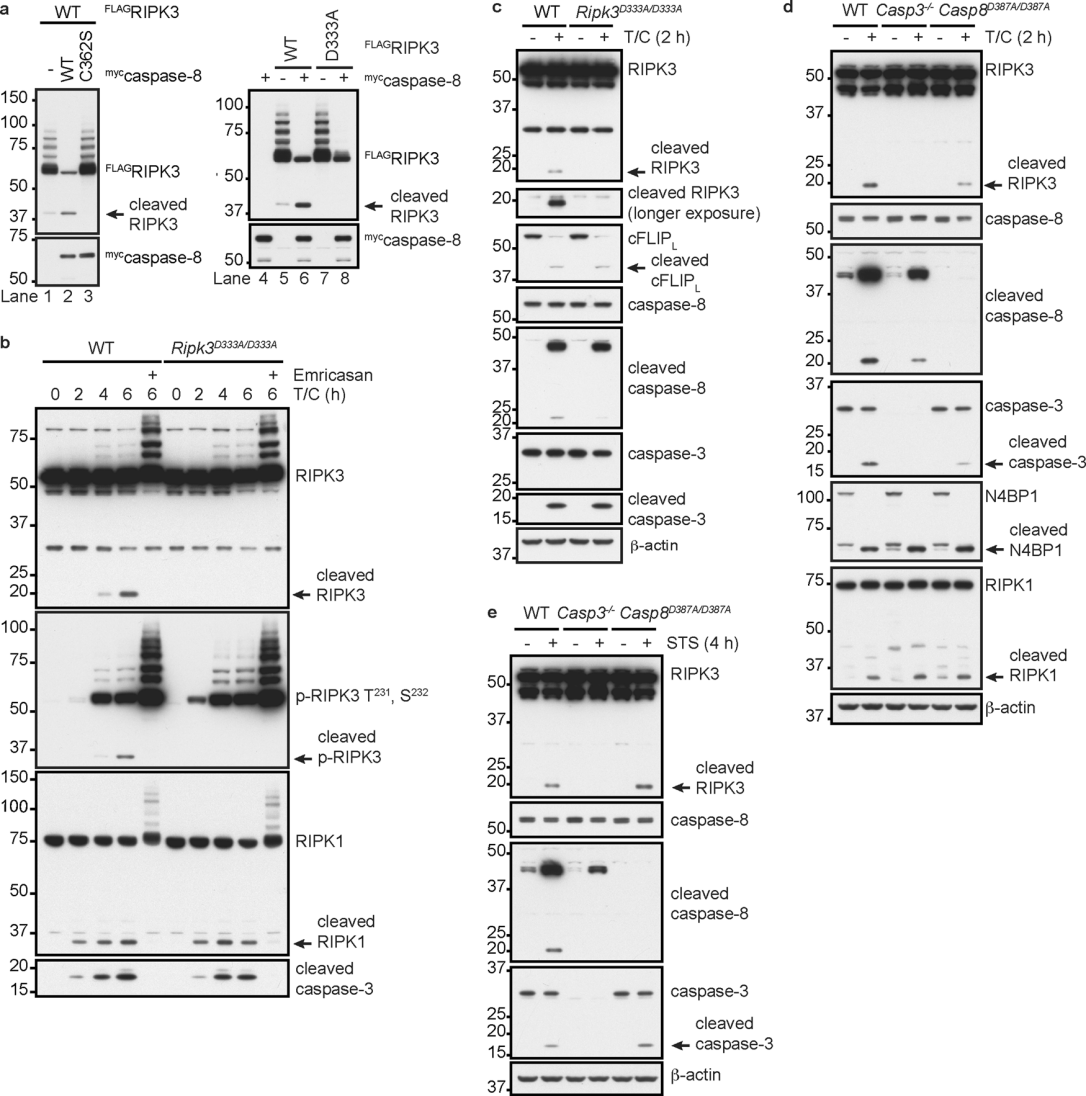
Cleavage of murine RIPK3 after Asp333. **a** Western blots of 293 T cells co-transfected with murine RIPK3 and caspase-8. **b** Western blots of MEFs treated with 100 ng/ml TNF (T), 10 μg/ml cycloheximide (C) and 20 μM emricasan, as indicated. **c**–**e** Western blots of BMDMs treated with 100 ng/ml TNF plus 5 μg/ml cycloheximide (T/C) or 1 μM staurosporine (STS). Results in (**a**–**e**) are each representative of two independent experiments.

Human caspase-8 is reported to cleave human RIPK3 after the sequence TEMD^328^, separating the N-terminal kinase domain from the C-terminal RIP homotypic interaction motif (RHIM) that mediates interactions with RIPK1, TRIF, or ZBP1 [45]. This cleavage site is conserved in mouse RIPK3, which has the sequence TEMD^333^. Consistent with cleavage of RIPK3 at this site, 293 T cells transfected with Flag-tagged mouse RIPK3 appeared to contain a small amount of the 38 kDa N-terminal RIPK3 cleavage product (Fig. 1a, lanes 1 and 5). Co-transfection of mouse caspase-8 enhanced production of this RIPK3 fragment (Fig. 1a, lanes 2 and 6), but catalytically inactive caspase-8^C362S^ did not (Fig. 1a; lane 3). Importantly, mutant RIPK3^D333A^ did not yield the 38 kDa species, confirming Asp^333^ as the site of caspase-8-dependent RIPK3 cleavage (Fig. 1a; lanes 7 and 8). WT caspase-8 also limited what appears to be ubiquitination of RIPK3 (Fig. 1a), but the significance of this modification is unclear. RIPK3 bearing K48-linked polyubiquitin may undergo proteasomal degradation [50]. Consistent with these data, the pan-caspase inhibitor emricasan increased the amount of endogenous RIPK3 that was modified with M1- and K48-linked polyubiquitin in WT mouse embryo fibroblasts (MEFs) exposed to the pro-apoptotic stimulus of TNF plus cycloheximide (T/C) (Fig. 1b and S1a).

To investigate the physiological significance of RIPK3 cleavage at Asp333, we generated knock-in mice expressing RIPK3^D333A^. *Ripk3*^*D333A/D333A*^ mice were viable and born at the expected frequency (56 out of 224 pups from *Ripk3*^*D333A/+*^ parents were *Ripk3*^*D333A/D333A*^). Western blotting of WT and *Ripk3*^*D333A/D333A*^ MEFs or bone marrow-derived macrophages (BMDMs) showed that RIPK3^D333A^ was expressed to the same extent as WT RIPK3 (Fig. 1b, c). When treated with T/C to activate caspase-8, WT and *Ripk3*^*D333A/D333A*^ cells exhibited comparable cleavage of caspase-8, cFLIP_L_, RIPK1, or caspase-3, but cleaved RIPK3 was only detected in WT cells (Fig. 1b, c). The 17 kDa C-terminal RIPK3 cleavage fragment and full-length RIPK3 were detected with antibody 1G6 (raised against residues 310-478 of mouse RIPK3). We also detected the 36 kDa N-terminal RIPK3 cleavage fragment in T/C-treated MEFs using an antibody that recognizes autophosphorylated RIPK3 (p-RIPK3^T231, S232^) (Fig. 1b). Both the N- and C-terminal RIPK3 cleavage products were caspase-dependent because they were not detected in the presence of emricasan (Fig. 1b). RIPK3 cleavage was also less evident in caspase-8 deficient cells (Figure S1b).

To exclude RIPK3 cleavage by caspase-3 and -7 downstream of caspase-8, we treated BMDMs lacking caspase-3 and/or -7 with T/C (Fig. S1c). To our surprise, *Casp3*^*−/−*^ BMDMs exhibited normal cleavage of RIPK1 and N4BP1, but no RIPK3 cleavage (Fig. 1d and S1c). This result suggests that caspase-3 cleaves RIPK3, not caspase-8 or -7. T/C-induced caspase-8 cleavage was slightly reduced in *Casp3*^*−/−*^ BMDMs compared with WT BMDMs, consistent with caspase-3 being able to cleave caspase-8 [51]. However, as demonstrated using *Casp8*^*D387A/D387A*^ BMDMs, cleavage between the catalytic subunits of caspase-8 is dispensable for the cleavage of RIPK3, RIPK1, N4BP1, and caspase-3 (Fig. 1d).

To confirm that RIPK3 is a caspase-3 substrate, we treated BMDMs with staurosporine to activate caspase-3 via the intrinsic apoptosis pathway. RIPK3 cleavage was detected in WT and *Casp8*^*−/−*^ *Mlkl*^*−/−*^ BMDMs, but not *Casp3*^*−/−*^ BMDMs (Fig. 1e and S1d). Thus, caspase-3 can cleave RIPK3 independent of caspase-8. Interestingly, when we revisited murine RIPK3 cleavage in 293 T cells, co-transfected caspase-8 enhanced RIPK3 cleavage even in the absence of endogenous caspase-3 (Fig. S1e). Therefore, caspase-8 may cleave RIPK3 directly when both proteins are overexpressed, whereas under more physiologically relevant conditions, caspase-8 or -9 appears to activate caspase-3 to cleave RIPK3.

**Fig. 2:**
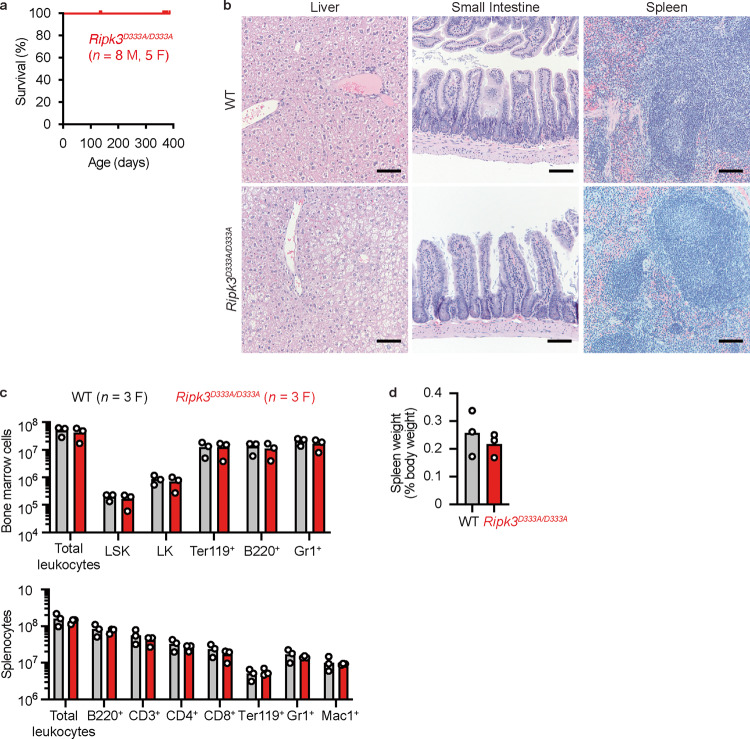
Cleavage of RIPK3 after Asp333 is dispensable for normal mouse development. **a** Survival curve of *Ripk3*^*D333A/D333A*^ mice. M, male. F, female. **b** Hematoxylin and eosin-stained tissue sections. Mice were aged 1 year. WT (*n* = 3 M, 2 F). *Ripk3*^*D333A/D333A*^ (*n* = 6 M, 3 F). Note that hepatocyte lipid vacuolization was increased in 5 out of 6 *Ripk3*^*D333A/D333A*^ males (a representative section is shown), but 0 out of 3 *Ripk3*^*D333A/D333A*^ females. This phenotype may reflect a sex difference rather than a genotype difference, because hepatocyte lipid vacuolization was also increased in 1 out of 3 WT males. **c** Leukocyte numbers in mice aged 9–14 weeks. **d** Spleen weight as a percentage of body weight of mice aged 1 year. Bars in (**c**) and (**d**) indicate the mean. Circles represent individual mice (WT, *n* = 3; *Ripk3*^*D333A/D333A*^, *n* = 3).

*Ripk3*^*D333A/D333A*^ mice aged for up to 1 year had no overt health problems (Fig. 2a). Histological analysis of the major organs did not reveal marked or consistent differences between WT and *Ripk3*^*D333A/D333A*^ siblings (Fig. 2b). The hematopoietic compartments of WT and *Ripk3*^*D333A/D333A*^ mice aged 9-14 weeks were comparable (Fig. 2c), and spleens from 1-year-old WT and *Ripk3*^*D333A/D333A*^ mice were comparable in size (Fig. 2d). Collectively, these data indicate that cleavage of RIPK3 at Asp333 is dispensable for normal mouse development.

### RIPK3^D333A^ does not increase sensitivity to TNF, poly(I:C), or LPS

**Fig. 3:**
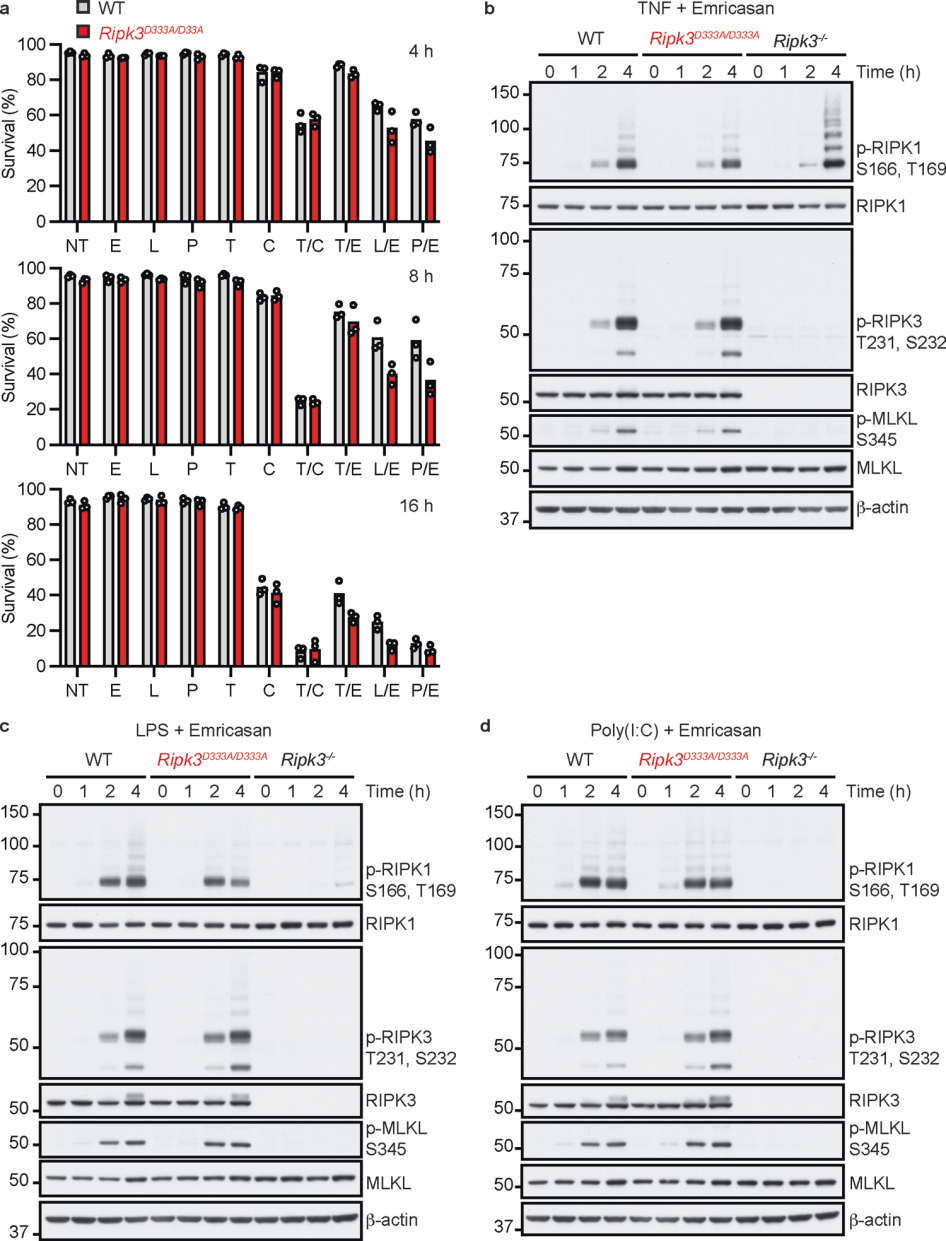
Impaired cleavage of RIPK3 does not sensitize BMDMs to TNF, poly(I:C), or LPS. **a** Graphs indicate the percentage of surviving BMDMs based on flow cytometry after staining with propidium iodide. Bars indicate the mean. Circles represent cells from different mice (WT, *n* = 3; *Ripk3*^*D333A/D333A*^, *n* = 3). NT, no treatment; E, emricasan; L, LPS; P, poly(I:C); T, TNF; C, cycloheximide. **b**–**d** Western blots of BMDMs. Results representative of 2 independent experiments.

TNF, poly(I:C), and LPS do not kill WT BMDMs in isolation, but induce necroptosis when combined with a pan-caspase inhibitor [12, 13, 52, 53]. We tested whether RIPK3^D333A^ bypassed the requirement for caspase inhibition, but *Ripk3*^*D333A/D333A*^ BMDMs, like WT BMDMs, remained viable after treatment with TNF, poly(I:C), or LPS (Fig. 3a and S2a–c). *Ripk3*^*D333A/D333A*^ BMDMs also activated MAPK and NF-κB signaling normally in response to TNF, poly(I:C), or LPS (Fig. S3a–c), and secreted normal amounts of cytokines and chemokines, including IL-6, CCL3, CCL5, CXCL1, CXCL2, and CXCL9 (Fig. S3d).

*Ripk3*^*D333A/D333A*^ BMDMs exposed to either the apoptotic stimulus of T/C, or the necroptotic stimulus of TNF plus emricasan (T/E), died at the same rate as WT BMDMs (Fig. 3a and S2a). *Ripk3*^*D333A/D333A*^ BMDMs exhibited a subtle, but reproducible acceleration in cell death between 4 and 8 h after treatment with LPS plus emricasan (L/E; Fig. 3a and S2b) or poly(I:C) plus emricasan (P/E; Fig. 3a and S2c). However, markers of necroptosis (p-RIPK1^S166, T169^, p-RIPK3^T231, S232^, and p-MLKL^S345^) were largely comparable in WT and *Ripk3*^*D333A/D333A*^ BMDMs within the first 4 h of T/E, L/E, or P/E treatment (Fig. 3b–d). Intriguingly, control *Ripk3*^*−/−*^ BMDMs revealed that autophosphorylation of RIPK1 Ser166 and Thr169 in response to L/E or P/E required RIPK3 (Fig. 3c and d). By contrast, T/E-induced RIPK1 autophosphorylation did not require RIPK3 (Fig. 3b), as reported previously for TNF-induced necroptosis [35]. These data suggest that RIPK1 recruitment to and/or oligomerization within TLR3 and TLR4 signaling complexes requires RIPK3. Although the RIPK1 scaffold is required for TLR3- or TLR4-induced TRIF-dependent NF-κB signaling [54, 55], RIPK3 was dispensable for poly(I:C)-induced MAPK and NF-κB signaling (Figure S4). Taken together, our data suggest that RIPK1 can be recruited directly to TRIF, but oligomerization and activation of its kinase activity is driven by interactions with RIPK3.

**Fig. 4:**
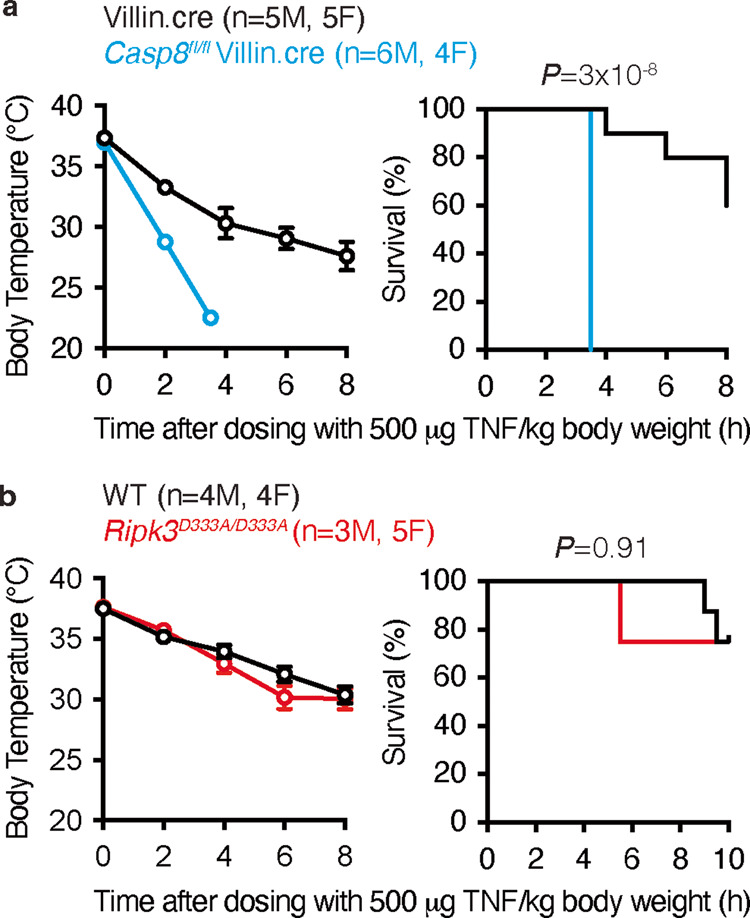
Caspase-8 deficiency in intestinal epithelial cells sensitizes mice to TNF toxicity, but RIPK3^D333A^ does not. **a**, **b** Body temperatures (mean ± sem) and Kaplan-Meier survival curves of 7- to 15-weeks-old littermates dosed with murine TNF. *P*-values determined by 2-sided Log-rank test. F female. M male.

Given that TNF toxicity in mice is mediated in part by RIPK3 [56, 57], we examined the response of *Ripk3*^*D333A/D333A*^ mice to high dose TNF. In contrast to mice lacking caspase-8 in intestinal epithelial cells, which were sensitized to TNF toxicity when compared with control mice (Fig. 4a), *Ripk3*^*D333A/D333A*^ mice exhibited TNF-induced hypothermia and morbidity comparable to that of WT mice (Fig. 4b). Therefore, caspase cleavage of RIPK3 is not required to suppress TNF toxicity in cells or mice.

### RIPK3^D333A^ does not alter sensitivity to MCMV-induced cell death

**Fig. 5:**
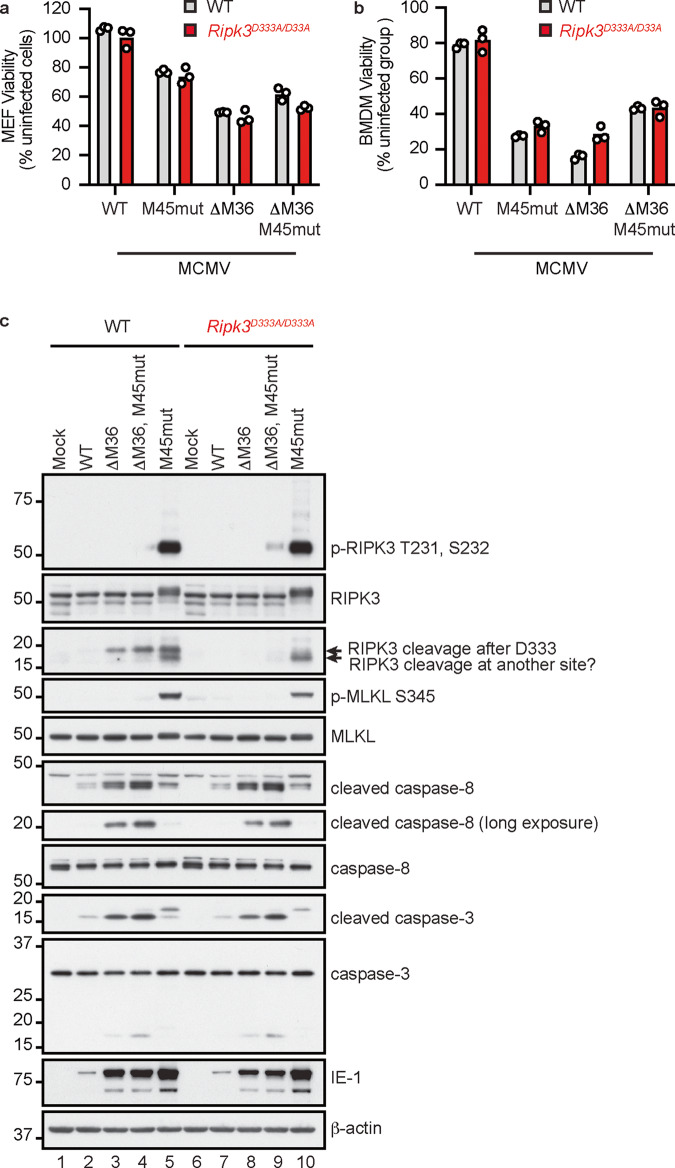
Effect of RIPK3^D333A^ on cell death induced by M45mutRHIM, ΔM36 or ΔM36M45 MCMV. **a**, **b** Graphs indicate MEF (**a**) or BMDM (**b**) viability after infection with MCMV for 18 h (moi = 5). Cell viability relative to uninfected cells was determined by Cell Titer Glo assay. Bars indicate the mean. Circles indicate technical replicates. Results are representative of 2 independent experiments. **c** Western blots of BMDMs infected with MCMV for 10 h (moi = 5).

To assess the effect of RIPK3^D333A^ on cell death mediated by ZBP1, we infected MEFs and BMDMs with WT and mutant forms of MCMV. ΔM36 MCMV induces apoptosis because it lacks the caspase-8 inhibitor M36 [58]. By contrast, M45mutRHIM MCMV promotes ZBP1- and RIPK3-dependent necroptosis because M45, the RHIM-bearing viral protein that inhibits RIPK1-RIPK3 or ZBP1-RIPK3 interactions, is disabled [59]. ΔM36, M45mutRHIM MCMV with both cell death inhibitors disabled has been shown to induce markers of apoptosis and necroptosis [60]. As expected, the mutant viruses induced more cell death in WT MEFs or BMDMs than the parental WT strain of MCMV (Fig. 5a, b, and S5). WT and *Ripk3*^*D333A/D333A*^ cells exhibited comparable cell death after infection with each of the viruses. Therefore, RIPK3 D333A does not overtly sensitize cells to MCMV-induced cell death.

Next, we analyzed the MCMV-infected cells for markers of cell death by western blotting. Expression of viral IE-1 confirmed successful infection of BMDMs (Fig. 5c). Markers of necroptosis (p-RIPK3^T231, S232^ and p-MLKL^S345^) and apoptosis (cleaved caspase-8 and cleaved caspase-3) were comparable between infected WT and *Ripk3*^*D333A/D333A*^ BMDMs, consistent with RIPK3 D333A not sensitizing to MCMV-induced cell death. As expected, infection with ΔM36 MCMV or ΔM36, M45mutRHIM MCMV (lanes 3, 4, 8, and 9) stimulated more robust cleavage of caspase-8 and -3 than WT or M45mutRHIM MCMV (lanes 2, 5, 7, and 10). However, what appeared to be the 17 kDa RIPK3 cleavage product (because it was detected in WT but not *Ripk3*^*D333A/D333A*^ BMDMs) was detected after infection with ΔM36 MCMV, ΔM36, M45mutRHIM MCMV, or M45mutRHIM MCMV (compare lanes 3, 4 and 5 with lanes 8, 9, and 10). M45mutRHIM MCMV infection also produced a slightly smaller RIPK3 species in both WT and *Ripk3*^*D333A/D333A*^ BMDMs (lanes 5 and 10). These findings suggest that a protease(s) other than caspase-3 or -8 may cleave RIPK3 in cells infected with M45mutRHIM MCMV.

## Discussion

The proteolytic activity of caspase-8 suppresses necroptosis in many cell types, including murine endothelial cells, macrophages, and T cells [9, 11, 14, 15]. Caspase-8 does this without inducing apoptosis, presumably because there is limited and/or transient activation of caspase-8. Caspase-8 cleavage of RIPK1, in particular, appears to destabilize the death signaling complex so that neither necroptosis nor apoptosis is triggered. Hence, mice expressing caspase-8 non-cleavable RIPK1^D325A^ exhibit aberrant caspase-8-dependent apoptosis and embryonic lethality [14, 29, 30, 49]. RIPK3 cleavage by caspase-8 has been reported [45], but its role in necroptosis suppression was unclear. We now show that endogenous RIPK3 cleavage in mouse cells is mediated by caspase-3 downstream of caspase-8 or -9. Indeed, we have only detected RIPK3 cleavage in cells given an apoptotic stimulus. Caspase-3 is dispensable for necroptosis suppression during embryogenesis because *Casp3*^*−/−*^ C57BL/6 mice are viable [61]. Therefore, it is perhaps unsurprising that *Ripk3*^*D333A/D333A*^ mice were born at the expected frequency and had a normal lifespan.

The only phenotype of note in our analyses of *Ripk3*^*D333A/D333A*^ cells was a subtle acceleration of P/E- or L/E-induced cell death. In P/E- or L/E-treated BMDMs, RIPK1 autophosphorylation was dependent on RIPK3, implying that RIPK3 promotes oligomerization of RIPK1 within the TRIF death signaling complex. Activation of RIPK1 is required for TLR3- or TLR4-induced necroptosis in BMDMs [12, 13, 62], but if it occurs downstream of RIPK3, then this might explain why the TRIF death signaling complex is slightly more sensitive to mutation of RIPK3 Asp333 than the TNFR1 death signaling complex. However, in the absence of detectable RIPK3 cleavage in this context, it is difficult to know whether RIPK3^D333A^ is preventing RIPK3 cleavage or just tweaking the structure of the RIPK3 scaffold.

We infected cells with MCMV to assess whether RIPK3 cleavage might impact ZBP1-driven cell death. M45mutRHIM MCMV triggers ZBP1-dependent necroptosis [59], whereas TNF-driven apoptosis is thought to contribute to the death of cells infected with ΔM36 MCMV [58]. ΔM36,M45mutRHIM MCMV might also engage the cell death machinery by other means because the virus remains attenuated in mice lacking both ZBP1 and TNF [60]. Therefore, we cannot be certain that the RIPK3 cleavage in WT MEFs infected with ΔM36,M45mut RHIM MCMV is dependent on ZBP1. Regardless of the upstream trigger, RIPK3^D333A^ prevented the appearance of the 17 kDa RIPK3 cleavage fragment. There were two RIPK3 cleavage products in cells infected with M45mut RHIM MCMV, one dependent on the Asp333 site and a smaller fragment that was not. It is unclear what produced the smaller RIPK3 fragment. Processing of caspase-3 and -8 was reduced in cells infected with M45mut RHIM MCMV when compared to cells infected with ΔM36,M45mut RHIM MCMV, presumably owing to caspase-8 inhibition by M36. Whether another caspase cleaves RIPK3 at Asp333 in cells infected with M45mut RHIM MCMV is unclear. MCMV infection of macrophages has been shown to trigger AIM2-dependent activation of caspase-1 [ref. [63]], but whether caspase-1 can cleave RIPK3 directly is unknown. We have observed what appears to be caspase-1-dependent cleavage of RIPK1 and RIPK3 in the intestines of *Casp8*^*C362A/C362A*^ *Mlkl*^*−/−*^ embryos [64]. Regardless, preventing cleavage of RIPK3 at Asp333 did not cause a marked increase in MCMV-induced cell death.

In sum, we can demonstrate caspase-3-dependent RIPK3 cleavage in apoptotic cells, but to date have been unable to detect RIPK3 cleavage in viable cells demonstrating caspase-8-dependent cleavage of N4BP1. Given that *Ripk3*^*D333A/D333A*^ mice are viable, we conclude that cleavage of other caspase-8 substrates, including RIPK1, must be sufficient to hold necroptosis in check.

## Materials and Methods

### Mice

Mouse studies complied with relevant ethics regulations and were approved by the Genentech institutional animal care and use committee. *Ripk3*^*−/−*^ mice [52] were on a C57BL/6 N background. *Villin*.cre transgenic mice [65] were on a C57BL/6 J background. Casp8^*fl/fl*^ mice were generated using C57BL/6 N C2 ES cells. *loxP* sites flanked the region corresponding to chr1:58,866,044-58,866,744 (NCBI GRCm39 assembly, annotation release 109), which contains exon 4. Genotyping primers 5’CAG TGG ATG CAT GCA AGG, 5’TCT CCT GAA GAT GGG TCA CC, and 5’GGA GCC AGT ATT CTG CTA GCA A amplified 349 bp WT, 435 bp cKO, and 304 bp KO DNA fragments.

*Ripk3*^*D333A/D333A*^ mice were generated by injecting C57BL/6 N zygotes with Cas9 mRNA, a sgRNA targeting 5’GCA CAG AAA TGG ATT GCC CG, and PAM AGG, and an oligonucleotide template (5’TCC AGG AAC TGG TCC GGA GGG TTC CTC CAA ATG CAG GCG GTC CAG CAT TTT AGA AAC CAT GGT TTC tCT gGG aCA ggC CAT TTC TGT GCC TCT TTG GCT TGG CTC TCT GGC AGA CAA GTT TCT GCC GCT GCT TCT GTG CTG AGA CAG ATA ATG) to mutate codons GAT (-Asp^333^-) to GCC (-Ala^333^-). Genotyping primers 5’AGT CTT GCC AGT GTC T, 5’AAA TGG ATT GCC CGA G, and 5’GCC TGT CCC AGA GAA amplified 276 bp WT and 271 bp *Ripk3*^*D333*A^ DNA fragments.

Littermates were dosed intravenously with 500 μg murine TNF (Genentech) per kg body weight. Mice were euthanized if severely lethargic or their body temperature dropped to 25 °C. For timed pregnancies, mice were designated E0.5 on the morning a vaginal plug was detected. Histological analyses were performed on tissues from unchallenged, 1-year-old mice. Serum cytokines and chemokines were measured using a mouse luminex panel (Millipore-Sigma, Burlington, MA, USA).

### Cells

Primary macrophages were differentiated from bone marrow cells in non-treated plates in the high glucose version of Dulbecco’s Modified Eagle Medium (DMEM) supplemented with 10% heat inactivated-fetal bovine serum, 2 mM glutamine, 50 μM 2-mercaptoethanol, 10 mM HEPES (pH 7.2), 1× non-essential amino acids solution, 100 U/ml penicillin, and 100 μg/ml streptomycin, and 50 ng/ml M-CSF (R&D Systems, Minneapolis, MN, USA) for 5–6 days. For survival assays, BMDMs were trypsinized and replated in 96-well dishes (5 × 10^4^ cells/well) in the same medium. The next day cells were treated with 100 ng/ml murine TNF (R&D Systems), 100 ng/ml ultra-pure LPS (Invivogen, San Diego, CA, USA), or 50 μg/ml low molecular weight poly(I:C) (Invivogen) either alone, or in combination with 20 μg/ml cycloheximide (Sigma-Aldrich, St. Louis, MO, USA), or 10 μM emricasan (Selleck Chemicals, Houston, TX, USA). In Fig. S2, BMDM viability was assessed after YOYO-1 (Molecular Probes, Eugene, OR, USA) staining and live-cell imaging in an Incucyte S3 Live-Cell Analysis System (Essen BioScience, Ann Arbor, MI, USA). In Fig. 3a, adherent BMDMs were trypsinized and pooled with non-adherent cells. Pooled cells were stained with propidium iodide (PI). The percentage of live, PI-negative cells was determined by flow cytometry.

MEFs were prepared by trypsin digestion of E14.5 embryo carcasses after removing the head and internal organs. Cells were plated on dishes pre-coated with 0.1% gelatin in PBS and grown in high glucose DMEM supplemented with 10% heat inactivated-fetal bovine serum, 50 μM 2-mercaptoethanol, 2 mM glutamine, 10 mM HEPES (pH 7.2), 1× non-essential amino acids solution, 100 U/ml penicillin, and 100 μg/ml streptomycin. MEFs were passaged no more than 5 times.

Human 293 T cells (ATCC CRL-3216, mycoplasma negative, but not authenticated) were transfected for 18 h with mouse *Ripk3* in pCMV-3tag6 (Agilent Technologies, Santa Clara, CA, USA) and mouse *caspase-8* in pCMV-3tag7 (Agilent Technologies) using FuGENE HD (Promega, Madison, WI, USA).

For gene deletion, we used Alt-R Cas9 (IDT, Coralville, IA, USA) and gene-specific sgRNAs (IDT). BMDMs were electroporated using nucleofector solution P3 and program CM-137 (Lonza, Morristown, NJ, USA). For 293 T cells, we used solution SF and program CM-130 (Lonza). BMDMs were maintained for an additional 5 days before further treatments. 293 T cells were cultured for a minimum of one week after electroporation. Target sequences were in mouse *Casp7* (CAT CAT CGA GCT CCG TCC CT and GGA CGG TTA CTT CAA AAC CC) and human *CASP3* (GAT CGT TGT AGA AGT CTA AC, GGA AGC GAA TCA ATG GAC TC, and CGT GGT ACA GAA CTG GAC TG). Negative control crRNAs (IDT) were annealed to Alt-R tracrRNA (IDT) prior to electroporation.

### Virus Infections

K181-BAC as well as K181-derived M45mutRHIM, ΔM36, and ΔM36/M45mutRHIM viruses were propagated in NIH 3T3 cells (ATCC CRL-1658, neither mycoplasma tested nor authenticated) as described [60, 66]. Cells were infected for 1 h (multiplicity of infection, moi = 5), and then the inoculate was replaced with fresh medium.

### Western blots and immunoprecipitations

Cells in Figs. 1a–e, S1b–e, S3a–c, and S4 were lysed in 20 mM Tris.HCl pH 7.5, 135 mM NaCl, 1.5 mM MgCl_2_, 1 mM EGTA, 1% Triton X-100, 10% glycerol, phosSTOP phosphatase inhibitor (Millipore-Sigma), and Halt protease inhibitor cocktail (Thermo Fisher Scientific, Waltham, MA, USA). Cells in Figs. 3b–d and 5c were lysed in 10 mM Tris-HCl pH 7.5, 150 mM NaCl, 2.5 mM MgCl_2_, 0.5 mM CaCl_2_, 1% NP40, phosSTOP phosphatase inhibitor, Halt protease inhibitor cocktail, and DNase (approximately 80 U/ml; Qiagen, Germantown, MD, USA).

For immunoprecipitations (IPs), 1% Triton X-100 lysis buffer was supplemented with 6 M urea and 10 mM N-ethylmaleimide. Lysates were diluted to 3 M urea prior to IP of K48- and K63-linked ubiquitin chains. Genentech antibodies binding to M1- (1F11/3F5/Y102L), K63- (Apu3.A8), and K48-linked chains (Apu2.07) were captured with Protein A Dynabeads (Thermo Fisher Scientific) at room temperature.

Genentech antibodies were used to immunoblot cFLIP (2.21H2), RIPK1 (10C7), p-RIPK1^S166, T169^ (GEN175-DP-B1), RIPK3 (1G6), and p-RIPK3^T231, S232^ (GEN135-35-9). Cell Signaling Technology (Danvers, MA, USA) antibodies detected cleaved caspase-8 (8952), caspase-3 (9662), cleaved caspase-3 (9664), caspase-7 (9492), cleaved caspase-7 (9491), PARP (9542), RIPK1 (3493), RIPK3 (15828), p-ERK (9101), ERK (9102), p-JNK (4668), JNK (9258), p-IκBα (2859), IκBα (9242), p-RelA (3033), RelA (8242), p-p38 (9211), and p38 (8690). Millipore-Sigma antibodies detected MLKL (3H1), p-MLKL^S345^ (MABC1158), and FLAG (A8592). Other antibodies detected β-actin (MP Biomedicals, Solon, OH, USA; mouse clone C4), caspase-8 (1G12, Enzo Life Sciences, Farmingdale, NY, USA), MCMV 1E-1 (Croma101; gift from Stipan Jonjic, University of Rijeka), and myc (GTX21261, Genetex, Irvine, CA, USA).

Uncropped western blots are provided as Supplementary Data.

### Flow cytometry

Cells were labelled with antibodies from BD Biosciences (San Jose, CA, USA). APC-anti-c-Kit (553356), APC-Cy7-anti-CD3 (557596), BV421-anti-Gr-1 (562709), BV421-anti-IgM (562595), BV421-anti-Sca-1 (562729), FITC-anti-B220 (553088), V500-anti-B220 (561226), FITC-anti-CD3 (553061), FITC-anti-CD4 (553651) FITC-anti-CD5 (553021), FITC-anti-CD8 (553031), FITC-anti-Gr-1 (553127), FITC-anti-Mac-1 (553310), FITC-anti-TER-119 (557915), PE-anti-CD4 (553653), PE-anti-Mac-1 (553311), PE-Cy7-anti-CD62L (560516) were diluted in 2% normal rat serum and 1 μg/ml 2.4G2 anti-CD16/CD32 (553142). Leukocytes were identified by their forward scatter (FSC) and side scatter profiles. Dead cells that stained with 7-AAD (BD Biosciences), plus doublets, identified by their FSC-A versus FSC-W profiles, were excluded from analyses. Data were acquired using a BD FACSCantoII cytometer and BD FACSDiva 8.0, and analyzed with FlowJo 10.3.

### Statistics

No sample size calculations were performed. Tissues and cells from at least 3 animals per genotype were analyzed. TNF challenge studies used 8-10 mice/group because there is greater variability between WT controls in these experiments. There was no method of randomization, no blinding, and no samples or animals were excluded from analyses. Statistics were calculated using Prism 9.1.2.

### Supplementary information

Supplementary Figure 1

Supplementary Figure 2

Supplementary Figure 3

Supplementary Figure 4

Supplementary FIgure 5

Supplementary Figure 6

Reproducibility checklist

## Data Availability

The datasets generated during and/or analysed during the current study are available from the corresponding author on reasonable request. Mice and antibody reagents generated by Genentech are available under a material transfer agreement with Genentech.

## References

[1] Varfolomeev EE, Schuchmann M, Luria V, Chiannilkulchai N, Beckmann JS, Mett IL (1998). Targeted disruption of the mouse Caspase 8 gene ablates cell death induction by the TNF receptors, Fas/Apo1, and DR3 and is lethal prenatally. Immunity.

[2] Juo P, Kuo CJ, Yuan J, Blenis J (1998). Essential requirement for caspase-8/FLICE in the initiation of the Fas-induced apoptotic cascade. Curr Biol.

[3] Estornes Y, Toscano F, Virard F, Jacquemin G, Pierrot A, Vanbervliet B (2012). dsRNA induces apoptosis through an atypical death complex associating TLR3 to caspase-8. Cell Death Differ.

[4] Zinngrebe J, Rieser E, Taraborrelli L, Peltzer N, Hartwig T, Ren H (2016). LUBAC deficiency perturbs TLR3 signaling to cause immunodeficiency and autoinflammation. J Exp Med.

[5] Kuriakose T, Man SM, Malireddi RK, Karki R, Kesavardhana S, Place DE (2016). ZBP1/DAI is an innate sensor of influenza virus triggering the NLRP3 inflammasome and programmed cell death pathways. Sci Immunol.

[6] Nogusa S, Thapa RJ, Dillon CP, Liedmann S, Oguin TH, Ingram JP (2016). RIPK3 Activates Parallel Pathways of MLKL-Driven Necroptosis and FADD-Mediated Apoptosis to Protect against Influenza A Virus. Cell Host Microbe.

[7] Thapa RJ, Ingram JP, Ragan KB, Nogusa S, Boyd DF, Benitez AA (2016). DAI Senses Influenza A Virus Genomic RNA and Activates RIPK3-Dependent Cell Death. Cell Host Microbe.

[8] Kaiser WJ, Upton JW, Long AB, Livingston-Rosanoff D, Daley-Bauer LP, Hakem R (2011). RIP3 mediates the embryonic lethality of caspase-8-deficient mice. Nature.

[9] Oberst A, Dillon CP, Weinlich R, McCormick LL, Fitzgerald P, Pop C (2011). Catalytic activity of the caspase-8-FLIP(L) complex inhibits RIPK3-dependent necrosis. Nature.

[10] Alvarez-Diaz S, Dillon CP, Lalaoui N, Tanzer MC, Rodriguez DA, Lin A (2016). The Pseudokinase MLKL and the Kinase RIPK3 Have Distinct Roles in Autoimmune Disease Caused by Loss of Death-Receptor-Induced Apoptosis. Immunity.

[11] Ch’en IL, Tsau JS, Molkentin JD, Komatsu M, Hedrick SM (2011). Mechanisms of necroptosis in T cells. J Exp Med.

[12] He S, Liang Y, Shao F, Wang X (2011). Toll-like receptors activate programmed necrosis in macrophages through a receptor-interacting kinase-3-mediated pathway. Proc Natl Acad Sci USA.

[13] Kaiser WJ, Sridharan H, Huang C, Mandal P, Upton JW, Gough PJ (2013). Toll-like receptor 3-mediated necrosis via TRIF, RIP3, and MLKL. J Biol Chem.

[14] Newton K, Wickliffe KE, Dugger DL, Maltzman A, Roose-Girma M, Dohse M (2019). Cleavage of RIPK1 by caspase-8 is crucial for limiting apoptosis and necroptosis. Nature.

[15] Fritsch M, Gunther SD, Schwarzer R, Albert MC, Schorn F, Werthenbach JP (2019). Caspase-8 is the molecular switch for apoptosis, necroptosis and pyroptosis. Nature.

[16] Thome M, Schneider P, Hofmann K, Fickenscher H, Meinl E, Neipel F (1997). Viral FLICE-inhibitory proteins (FLIPs) prevent apoptosis induced by death receptors. Nature.

[17] Li M, Beg AA (2000). Induction of necrotic-like cell death by tumor necrosis factor alpha and caspase inhibitors: novel mechanism for killing virus-infected cells. J Virol.

[18] Skaletskaya A, Bartle LM, Chittenden T, McCormick AL, Mocarski ES, Goldmacher VS (2001). A cytomegalovirus-encoded inhibitor of apoptosis that suppresses caspase-8 activation. Proc Natl Acad Sci USA.

[19] Dufour F, Sasseville AM, Chabaud S, Massie B, Siegel RM, Langelier Y (2011). The ribonucleotide reductase R1 subunits of herpes simplex virus types 1 and 2 protect cells against TNFalpha- and FasL-induced apoptosis by interacting with caspase-8. Apoptosis.

[20] Fu TM, Li Y, Lu A, Li Z, Vajjhala PR, Cruz AC (2016). Cryo-EM Structure of Caspase-8 Tandem DED Filament Reveals Assembly and Regulation Mechanisms of the Death-Inducing Signaling Complex. Mol Cell.

[21] Fox JL, Hughes MA, Meng X, Sarnowska NA, Powley IR, Jukes-Jones R (2021). Cryo-EM structural analysis of FADD:Caspase-8 complexes defines the catalytic dimer architecture for co-ordinated control of cell fate. Nat Commun.

[22] Stennicke HR, Jurgensmeier JM, Shin H, Deveraux Q, Wolf BB, Yang X (1998). Pro-caspase-3 is a major physiologic target of caspase-8. J Biol Chem.

[23] Hughes MA, Harper N, Butterworth M, Cain K, Cohen GM, MacFarlane M (2009). Reconstitution of the death-inducing signaling complex reveals a substrate switch that determines CD95-mediated death or survival. Mol Cell.

[24] Keller N, Mares J, Zerbe O, Grutter MG (2009). Structural and biochemical studies on procaspase-8: new insights on initiator caspase activation. Structure.

[25] Oberst A, Pop C, Tremblay AG, Blais V, Denault JB, Salvesen GS (2010). Inducible dimerization and inducible cleavage reveal a requirement for both processes in caspase-8 activation. J Biol Chem.

[26] Lakhani SA, Masud A, Kuida K, Porter GA, Booth CJ, Mehal WZ (2006). Caspases 3 and 7: key mediators of mitochondrial events of apoptosis. Science.

[27] Boatright KM, Deis C, Denault JB, Sutherlin DP, Salvesen GS (2004). Activation of caspases-8 and -10 by FLIP(L). Biochem J.

[28] Yu JW, Jeffrey PD, Shi Y (2009). Mechanism of procaspase-8 activation by c-FLIPL. Proc Natl Acad Sci USA.

[29] Lalaoui N, Boyden SE, Oda H, Wood GM, Stone DL, Chau D (2020). Mutations that prevent caspase cleavage of RIPK1 cause autoinflammatory disease. Nature.

[30] Tao P, Sun J, Wu Z, Wang S, Wang J, Li W (2020). A dominant autoinflammatory disease caused by non-cleavable variants of RIPK1. Nature.

[31] Cho YS, Challa S, Moquin D, Genga R, Ray TD, Guildford M (2009). Phosphorylation-driven assembly of the RIP1-RIP3 complex regulates programmed necrosis and virus-induced inflammation. Cell.

[32] He S, Wang L, Miao L, Wang T, Du F, Zhao L (2009). Receptor interacting protein kinase-3 determines cellular necrotic response to TNF-alpha. Cell.

[33] Zhang DW, Shao J, Lin J, Zhang N, Lu BJ, Lin SC (2009). RIP3, an energy metabolism regulator that switches TNF-induced cell death from apoptosis to necrosis. Science.

[34] Murphy JM (2020). The Killer Pseudokinase Mixed Lineage Kinase Domain-Like Protein (MLKL). Cold Spring Harb Perspect Biol.

[35] Newton K, Wickliffe KE, Maltzman A, Dugger DL, Strasser A, Pham VC (2016). RIPK1 inhibits ZBP1-driven necroptosis during development. Nature.

[36] Takaoka A, Wang Z, Choi MK, Yanai H, Negishi H, Ban T (2007). DAI (DLM-1/ZBP1) is a cytosolic DNA sensor and an activator of innate immune response. Nature.

[37] Maelfait J, Liverpool L, Bridgeman A, Ragan KB, Upton JW, Rehwinkel J (2017). Sensing of viral and endogenous RNA by ZBP1/DAI induces necroptosis. EMBO J.

[38] Sridharan H, Ragan KB, Guo H, Gilley RP, Landsteiner VJ, Kaiser WJ (2017). Murine cytomegalovirus IE3-dependent transcription is required for DAI/ZBP1-mediated necroptosis. EMBO Rep.

[39] Guo H, Gilley RP, Fisher A, Lane R, Landsteiner VJ, Ragan KB (2018). Species-independent contribution of ZBP1/DAI/DLM-1-triggered necroptosis in host defense against HSV1. Cell Death Dis.

[40] Jiao H, Wachsmuth L, Kumari S, Schwarzer R, Lin J, Eren RO (2020). Z-nucleic-acid sensing triggers ZBP1-dependent necroptosis and inflammation. Nature.

[41] Wang R, Li H, Wu J, Cai ZY, Li B, Ni H (2020). Gut stem cell necroptosis by genome instability triggers bowel inflammation. Nature.

[42] Zhang T, Yin C, Boyd DF, Quarato G, Ingram JP, Shubina M (2020). Influenza Virus Z-RNAs Induce ZBP1-Mediated Necroptosis. Cell.

[43] Koehler H, Cotsmire S, Zhang T, Balachandran S, Upton JW, Langland J (2021). Vaccinia virus E3 prevents sensing of Z-RNA to block ZBP1-dependent necroptosis. Cell Host Microbe.

[44] Dillon CP, Weinlich R, Rodriguez DA, Cripps JG, Quarato G, Gurung P (2014). RIPK1 blocks early postnatal lethality mediated by caspase-8 and RIPK3. Cell.

[45] Feng S, Yang Y, Mei Y, Ma L, Zhu DE, Hoti N (2007). Cleavage of RIP3 inactivates its caspase-independent apoptosis pathway by removal of kinase domain. Cell Signal.

[46] Zhang H, Zhou X, McQuade T, Li J, Chan FK, Zhang J (2011). Functional complementation between FADD and RIP1 in embryos and lymphocytes. Nature.

[47] Kaiser WJ, Daley-Bauer LP, Thapa RJ, Mandal P, Berger SB, Huang C (2014). RIP1 suppresses innate immune necrotic as well as apoptotic cell death during mammalian parturition. Proc Natl Acad Sci USA.

[48] Rickard JA, O’Donnell JA, Evans JM, Lalaoui N, Poh AR, Rogers T (2014). RIPK1 regulates RIPK3-MLKL-driven systemic inflammation and emergency hematopoiesis. Cell.

[49] Zhang X, Dowling JP, Zhang J (2019). RIPK1 can mediate apoptosis in addition to necroptosis during embryonic development. Cell Death Dis.

[50] Moriwaki K, Chan FKM (2016). Regulation of RIPK3-and RHIM-dependent Necroptosis by the Proteasome. J Biol Chem.

[51] Slee EA, Harte MT, Kluck RM, Wolf BB, Casiano CA, Newmeyer DD (1999). Ordering the cytochrome c-initiated caspase cascade: hierarchical activation of caspases-2, -3, -6, -7, -8, and -10 in a caspase-9-dependent manner. J Cell Biol.

[52] Newton K, Dugger DL, Wickliffe KE, Kapoor N, de Almagro MC, Vucic D (2014). Activity of protein kinase RIPK3 determines whether cells die by necroptosis or apoptosis. Science.

[53] McComb S, Cessford E, Alturki NA, Joseph J, Shutinoski B, Startek JB (2014). Type-I interferon signaling through ISGF3 complex is required for sustained Rip3 activation and necroptosis in macrophages. Proc Natl Acad Sci USA.

[54] Meylan E, Burns K, Hofmann K, Blancheteau V, Martinon F, Kelliher M (2004). RIP1 is an essential mediator of Toll-like receptor 3-induced NF-kappa B activation. Nat Immunol.

[55] Cusson-Hermance N, Khurana S, Lee TH, Fitzgerald KA, Kelliher MA (2005). Rip1 mediates the Trif-dependent toll-like receptor 3- and 4-induced NF-kappaB activation but does not contribute to interferon regulatory factor 3 activation. J Biol Chem.

[56] Duprez L, Takahashi N, Van Hauwermeiren F, Vandendriessche B, Goossens V, Vanden Berghe T (2011). RIP kinase-dependent necrosis drives lethal systemic inflammatory response syndrome. Immunity.

[57] Newton K, Dugger DL, Maltzman A, Greve JM, Hedehus M, Martin-McNulty B (2016). RIPK3 deficiency or catalytically inactive RIPK1 provides greater benefit than MLKL deficiency in mouse models of inflammation and tissue injury. Cell Death Differ.

[58] Ebermann L, Ruzsics Z, Guzman CA, van Rooijen N, Casalegno-Garduno R, Koszinowski U (2012). Block of death-receptor apoptosis protects mouse cytomegalovirus from macrophages and is a determinant of virulence in immunodeficient hosts. PLoS Pathog.

[59] Upton JW, Kaiser WJ, Mocarski ES (2012). DAI/ZBP1/DLM-1 complexes with RIP3 to mediate virus-induced programmed necrosis that is targeted by murine cytomegalovirus vIRA. Cell Host Microbe.

[60] Daley-Bauer LP, Roback L, Crosby LN, McCormick AL, Feng Y, Kaiser WJ (2017). Mouse cytomegalovirus M36 and M45 death suppressors cooperate to prevent inflammation resulting from antiviral programmed cell death pathways. Proc Natl Acad Sci USA.

[61] Leonard JR, Klocke BJ, D’Sa C, Flavell RA, Roth KA (2002). Strain-dependent neurodevelopmental abnormalities in caspase-3-deficient mice. J Neuropathol Exp Neurol.

[62] Polykratis A, Hermance N, Zelic M, Roderick J, Kim C, Van TM (2014). Cutting edge: RIPK1 Kinase inactive mice are viable and protected from TNF-induced necroptosis in vivo. J Immunol.

[63] Rathinam VA, Jiang Z, Waggoner SN, Sharma S, Cole LE, Waggoner L (2010). The AIM2 inflammasome is essential for host defense against cytosolic bacteria and DNA viruses. Nat Immunol.

[64] Newton K, Wickliffe KE, Maltzman A, Dugger DL, Reja R, Zhang Y (2019). Activity of caspase-8 determines plasticity between cell death pathways. Nature.

[65] Madison BB, Dunbar L, Qiao XT, Braunstein K, Braunstein E, Gumucio DL (2002). Cis elements of the villin gene control expression in restricted domains of the vertical (crypt) and horizontal (duodenum, cecum) axes of the intestine. J Biol Chem.

[66] Upton JW, Kaiser WJ, Mocarski ES (2010). Virus inhibition of RIP3-dependent necrosis. Cell Host Microbe.

