# Supplementary figures and images for: Caspase cleavage of RIPK3 after Asp333 is dispensable for mouse embryogenesis
Source: Cell Death Differ. 2024 Jan 8;31(2):254–62. doi: 10.1038/s41418-023-01255-5 (PMC10850060; doi:10.1038/s41418-023-01255-5)

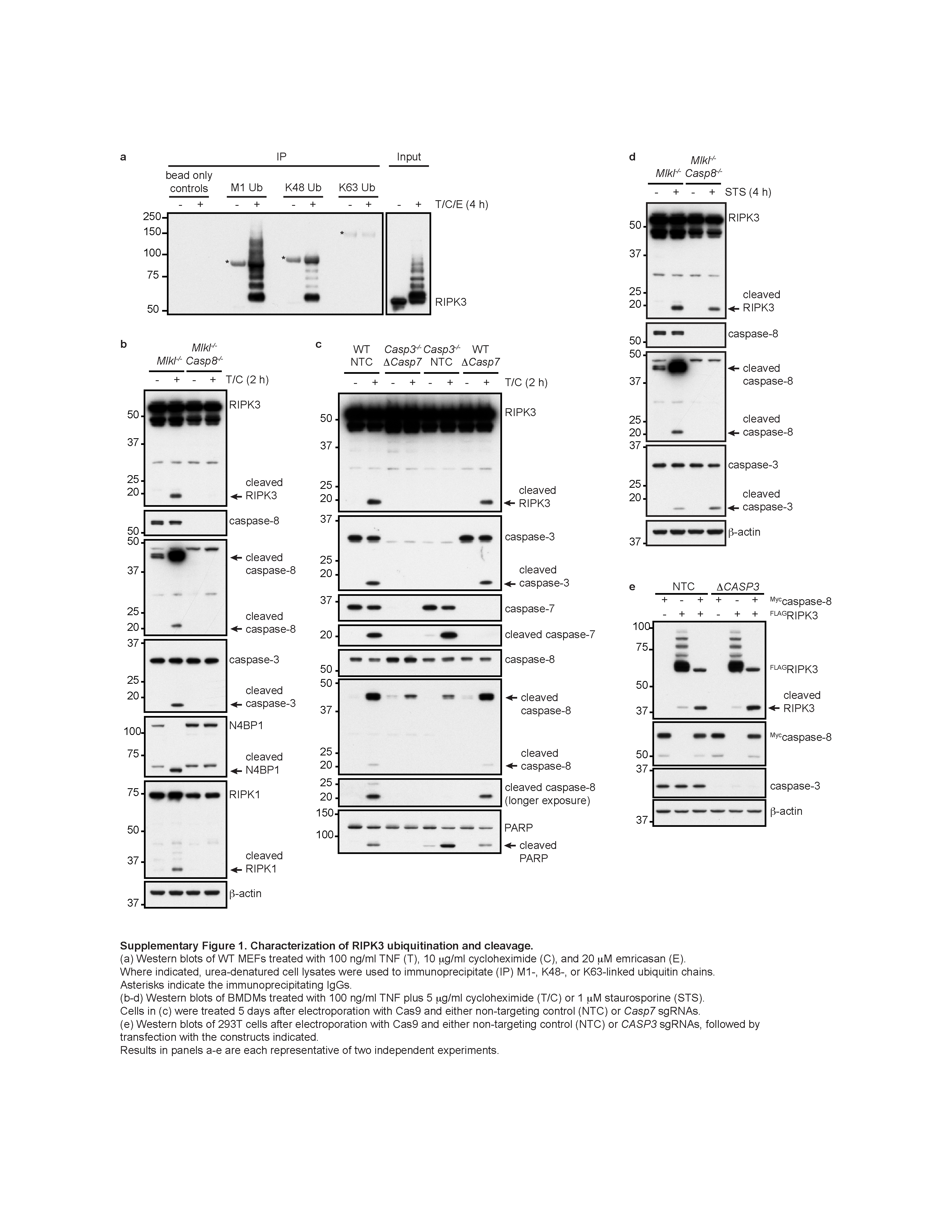

Supplement: Supplementary file 1 — Supplementary Figure 1 [file 41418_2023_1255_MOESM1_ESM.tif]

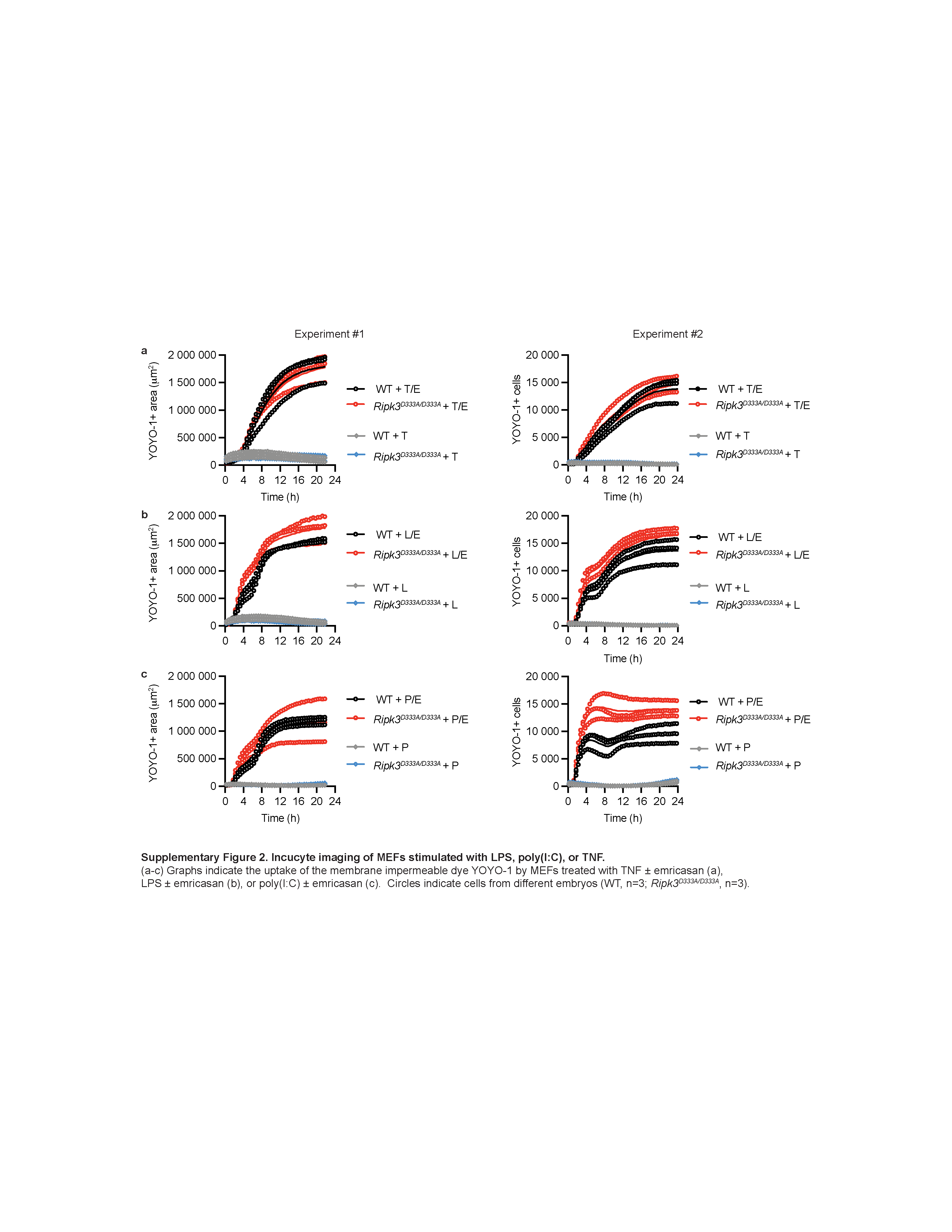

Supplement: Supplementary file 2 — Supplementary Figure 2 [file 41418_2023_1255_MOESM2_ESM.tif]

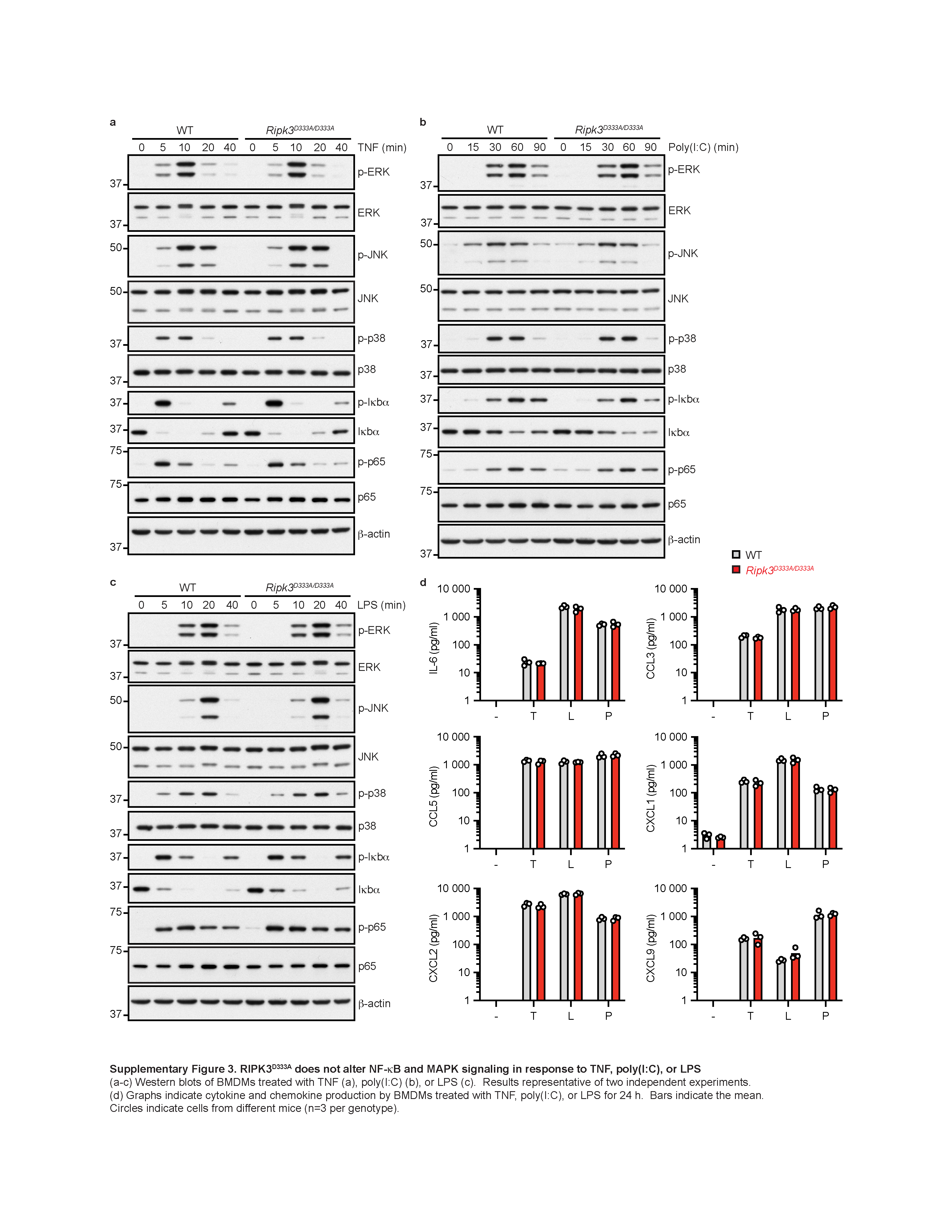

Supplement: Supplementary file 3 — Supplementary Figure 3 [file 41418_2023_1255_MOESM3_ESM.tif]

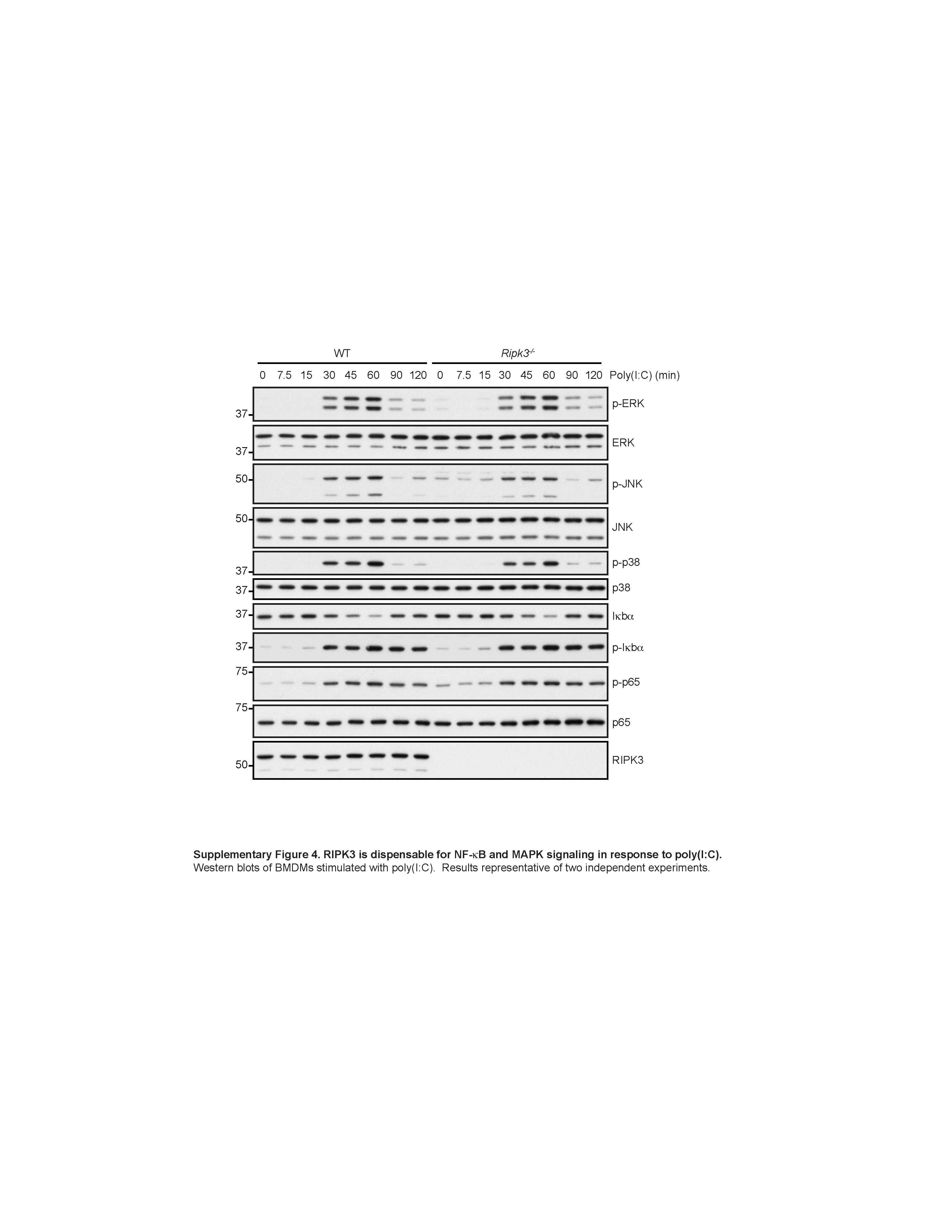

Supplement: Supplementary file 4 — Supplementary Figure 4 [file 41418_2023_1255_MOESM4_ESM.tif]

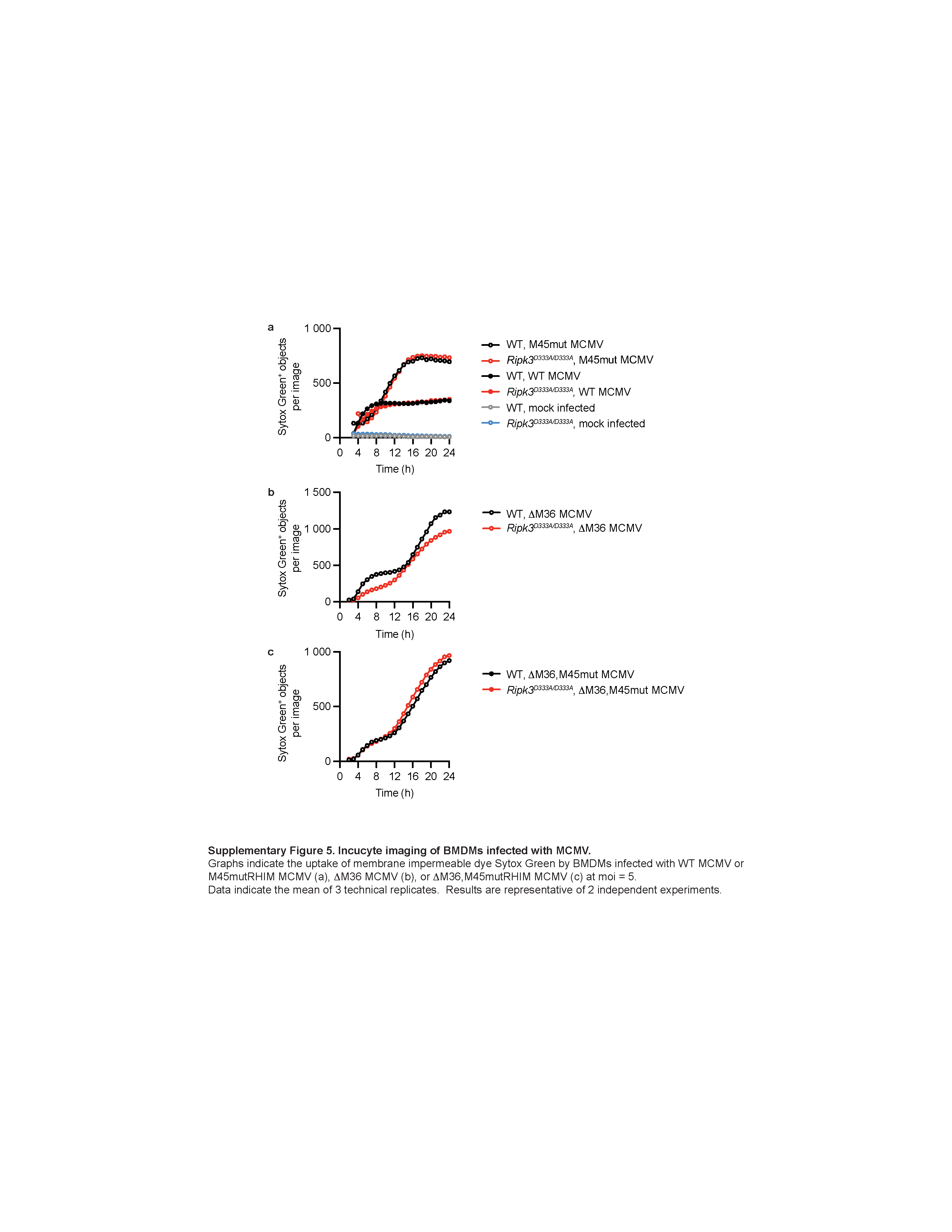

Supplement: Supplementary file 5 — Supplementary FIgure 5 [file 41418_2023_1255_MOESM5_ESM.tif]
