# Supplementary material for: Caspase cleavage of RIPK3 after Asp333 is dispensable for mouse embryogenesis
Source: Cell Death Differ. 2024 Jan 8;31(2):254–62. doi: 10.1038/s41418-023-01255-5 (PMC10850060; doi:10.1038/s41418-023-01255-5)

Figure 1a

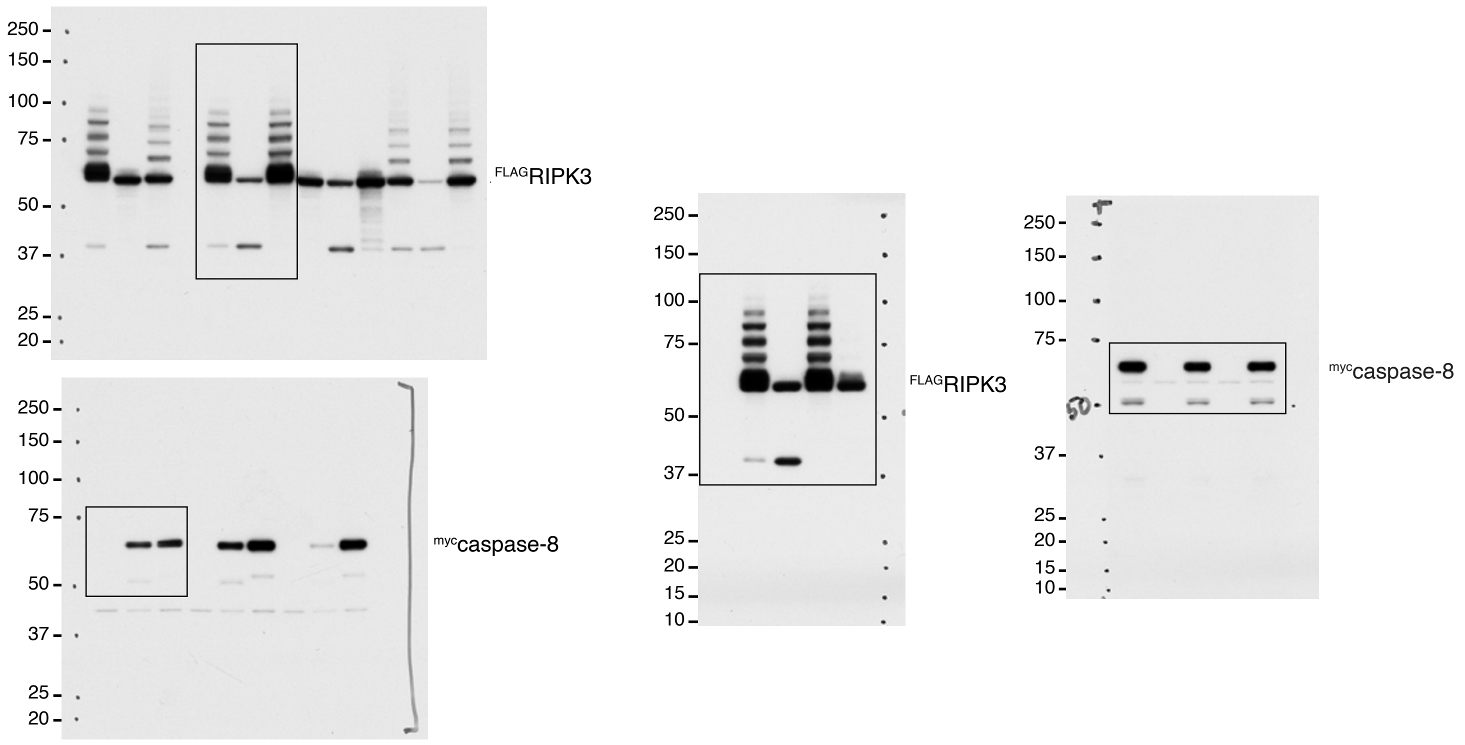

Figure 1b

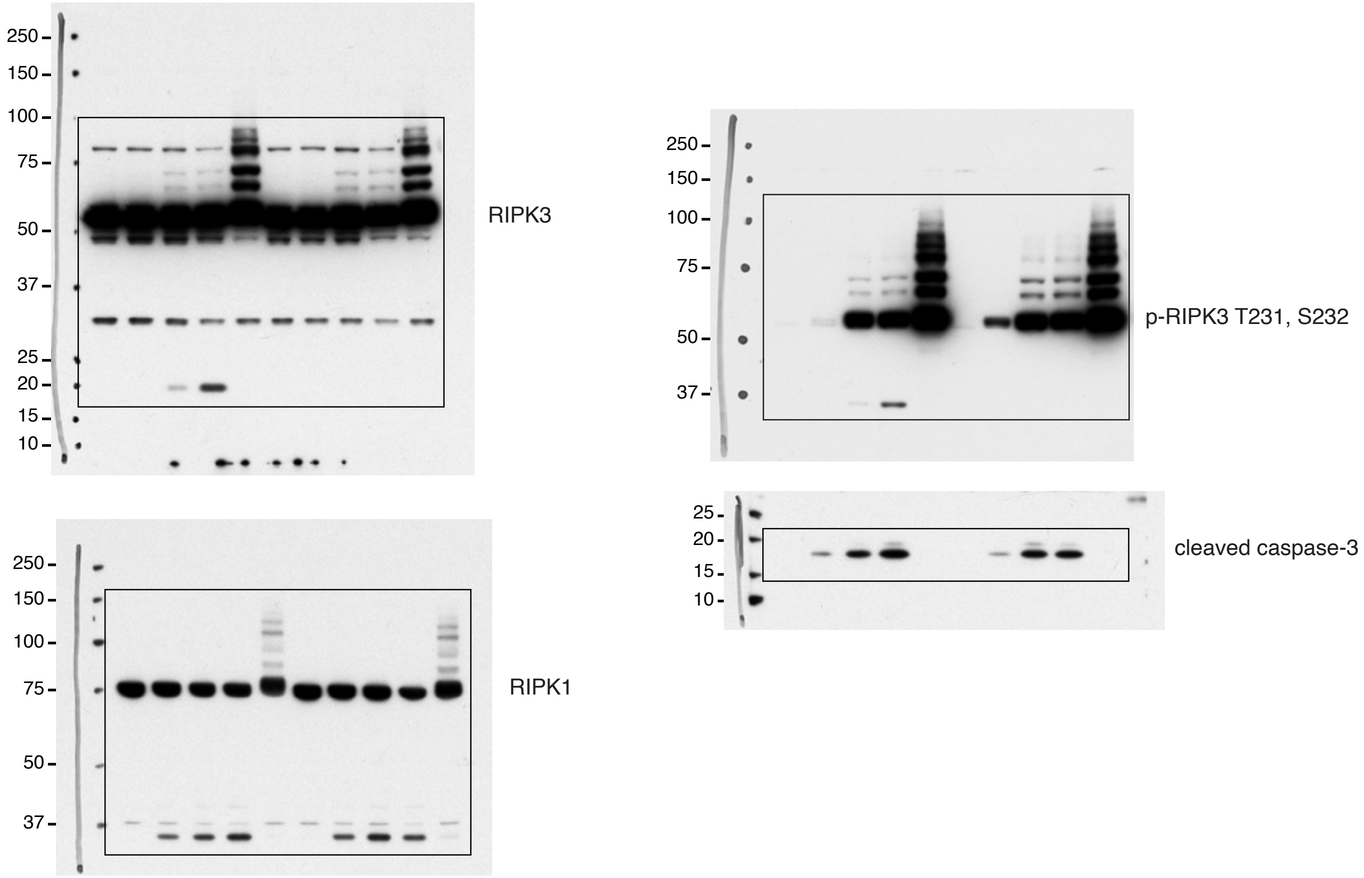

Figure 1c

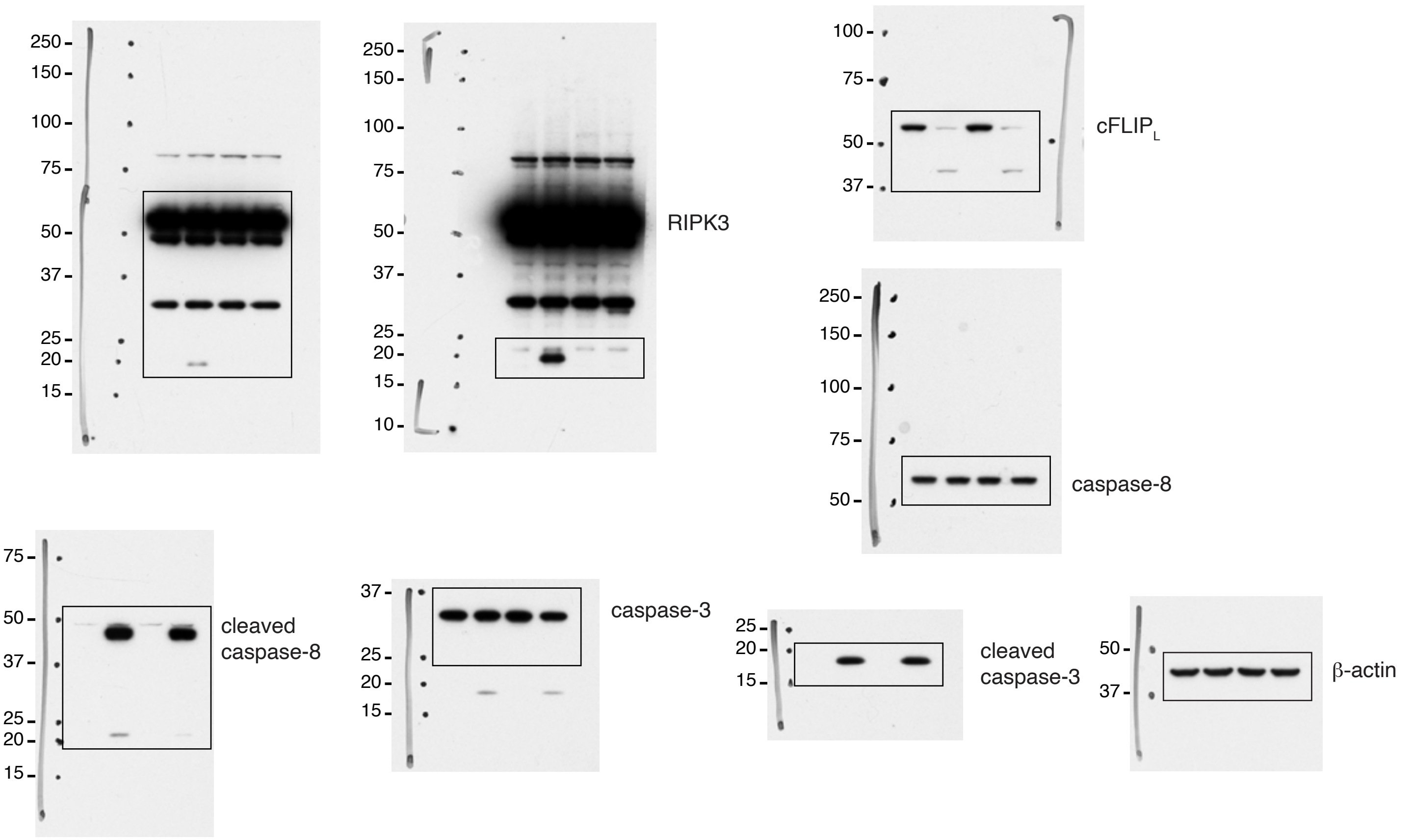

Figure 1d

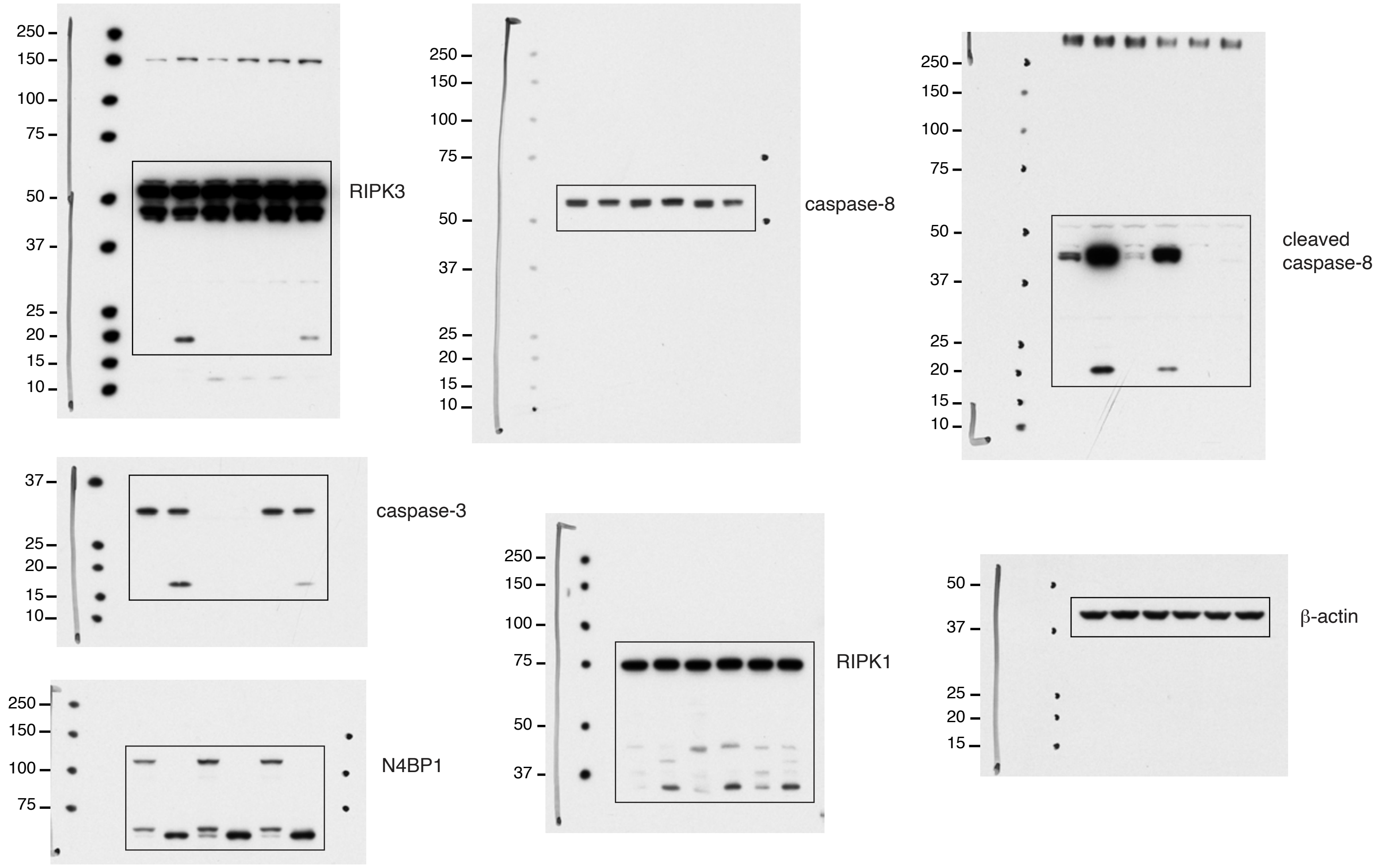

Figure 1e

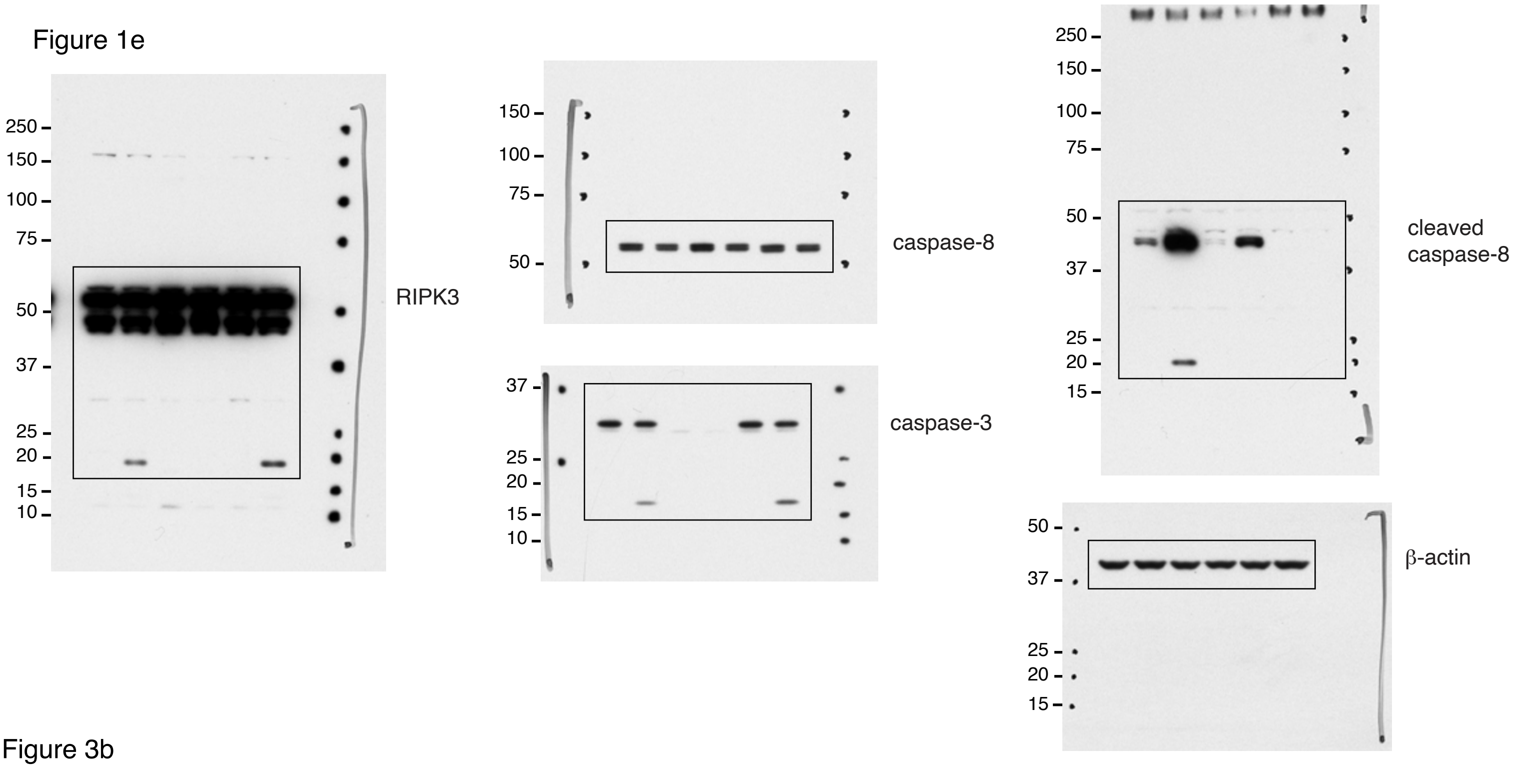

Figure 3b

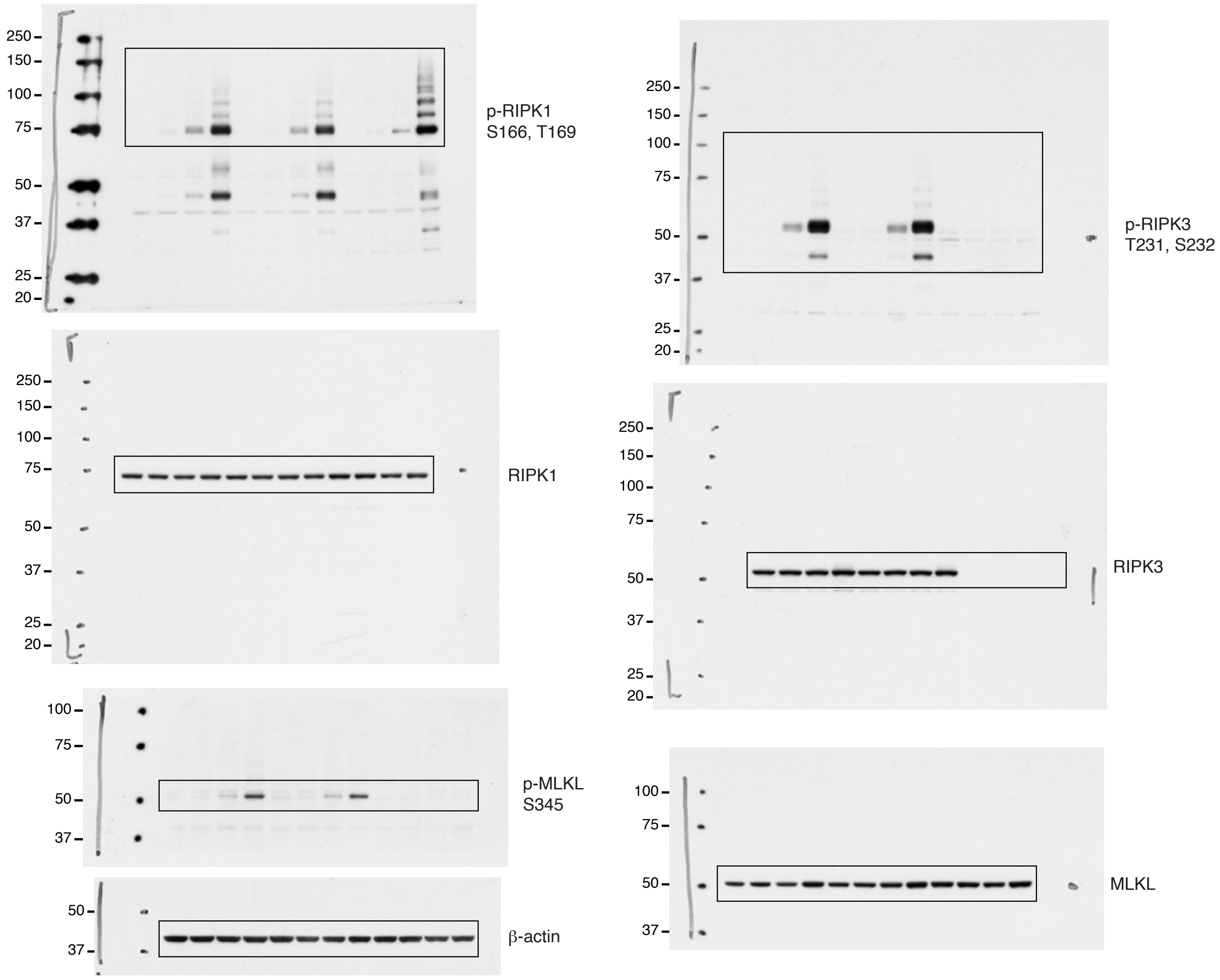

Figure 3c

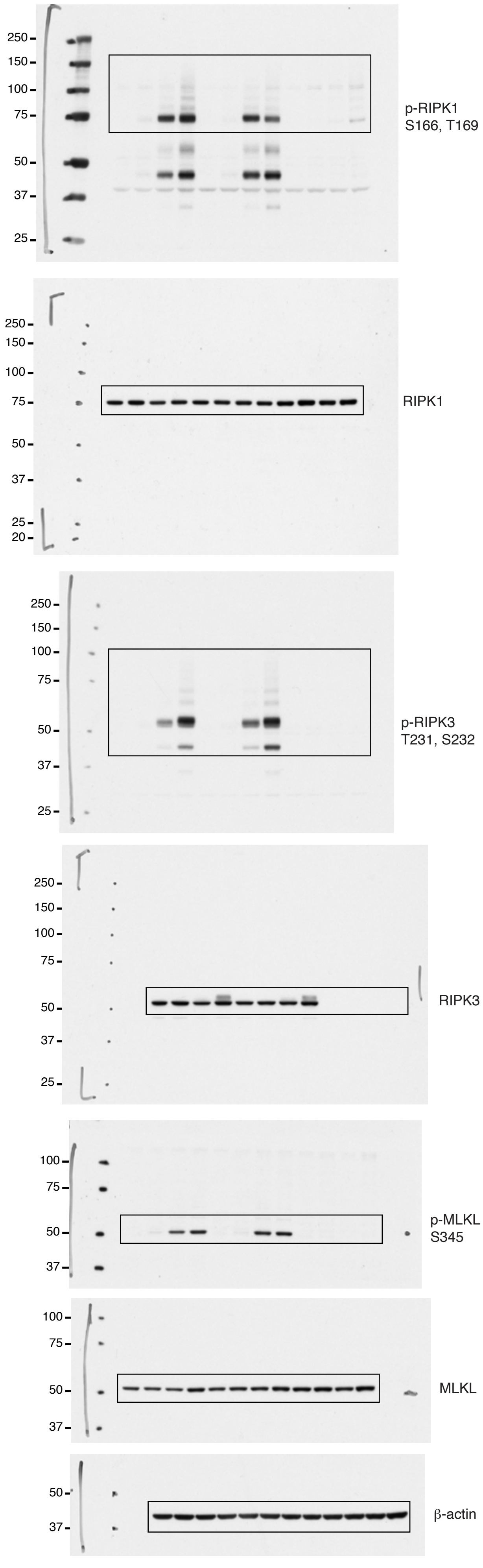

Figure 3d

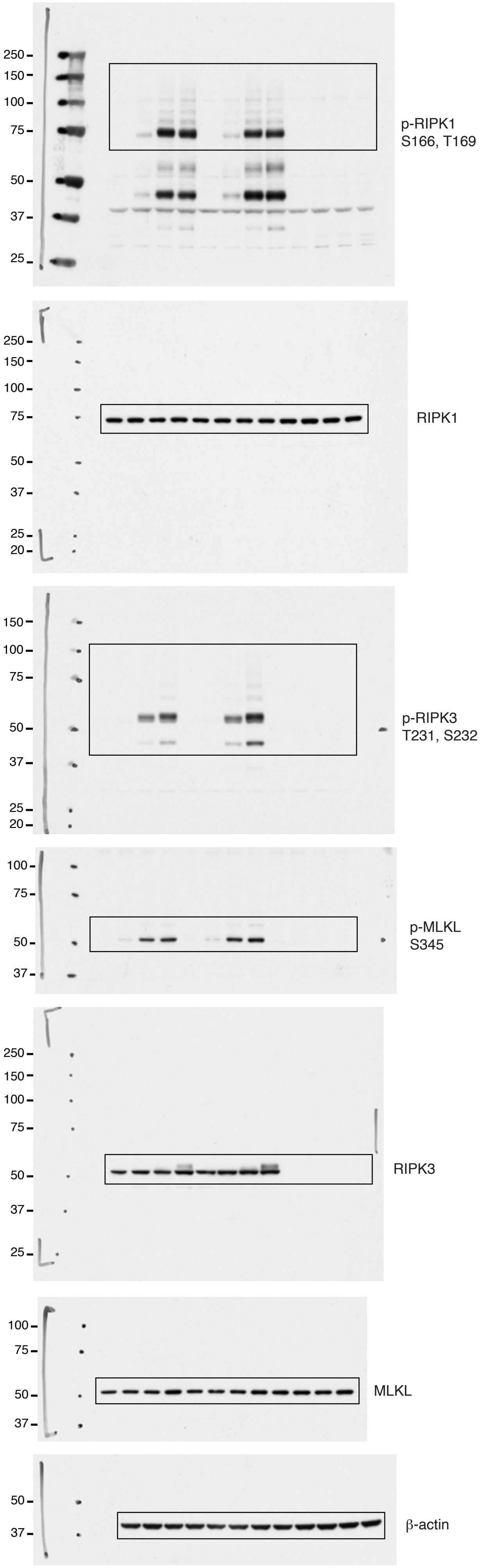

Figure 5c

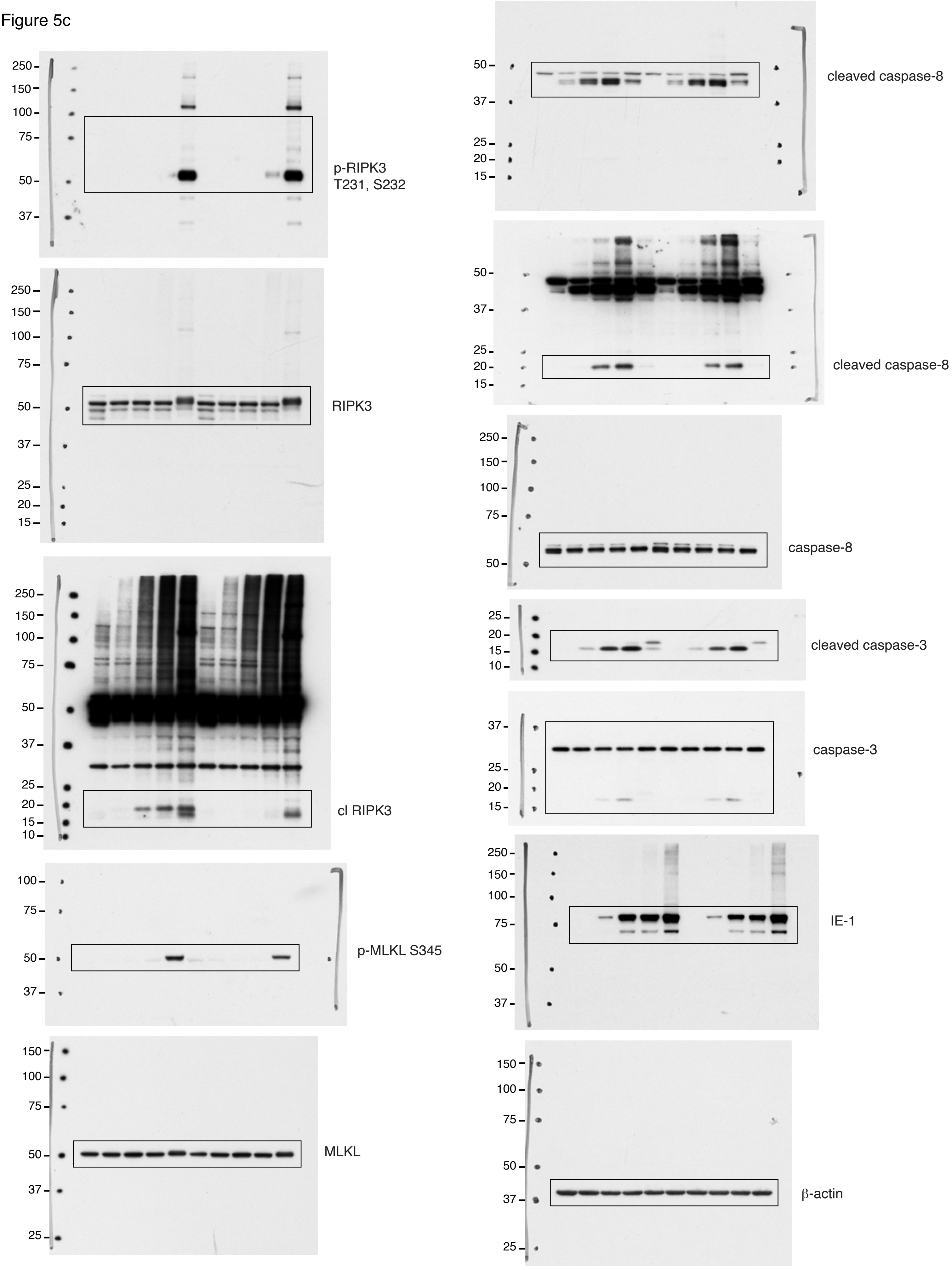
p-RIPK3
T231, S232
RIPK3
cl RIPK3
p-MLKL S345
MLKL
cleaved caspase-8
cleaved caspase-8
caspase-8
cleaved caspase-3
caspase-3
IE-1
β-actin

Figure S1a

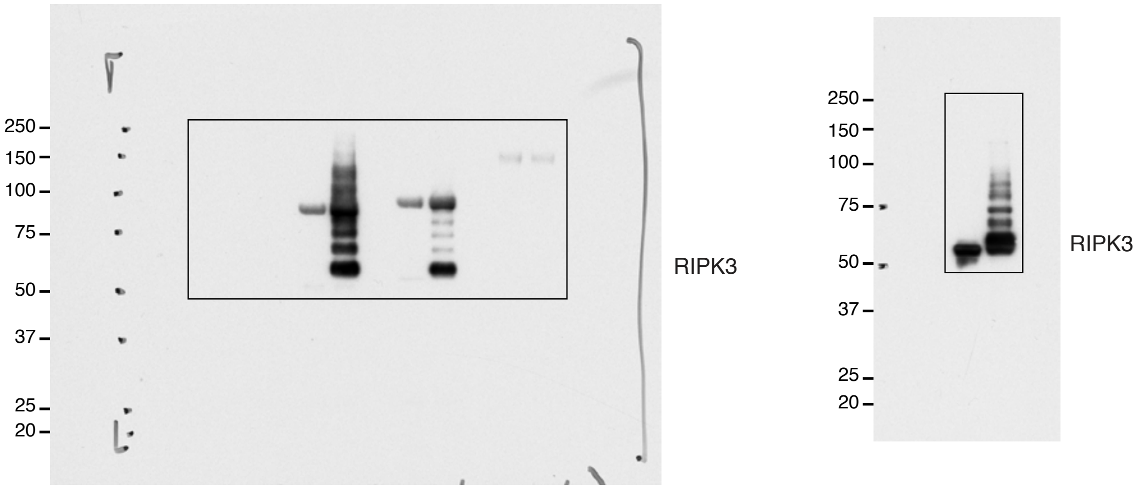

Figure S1b

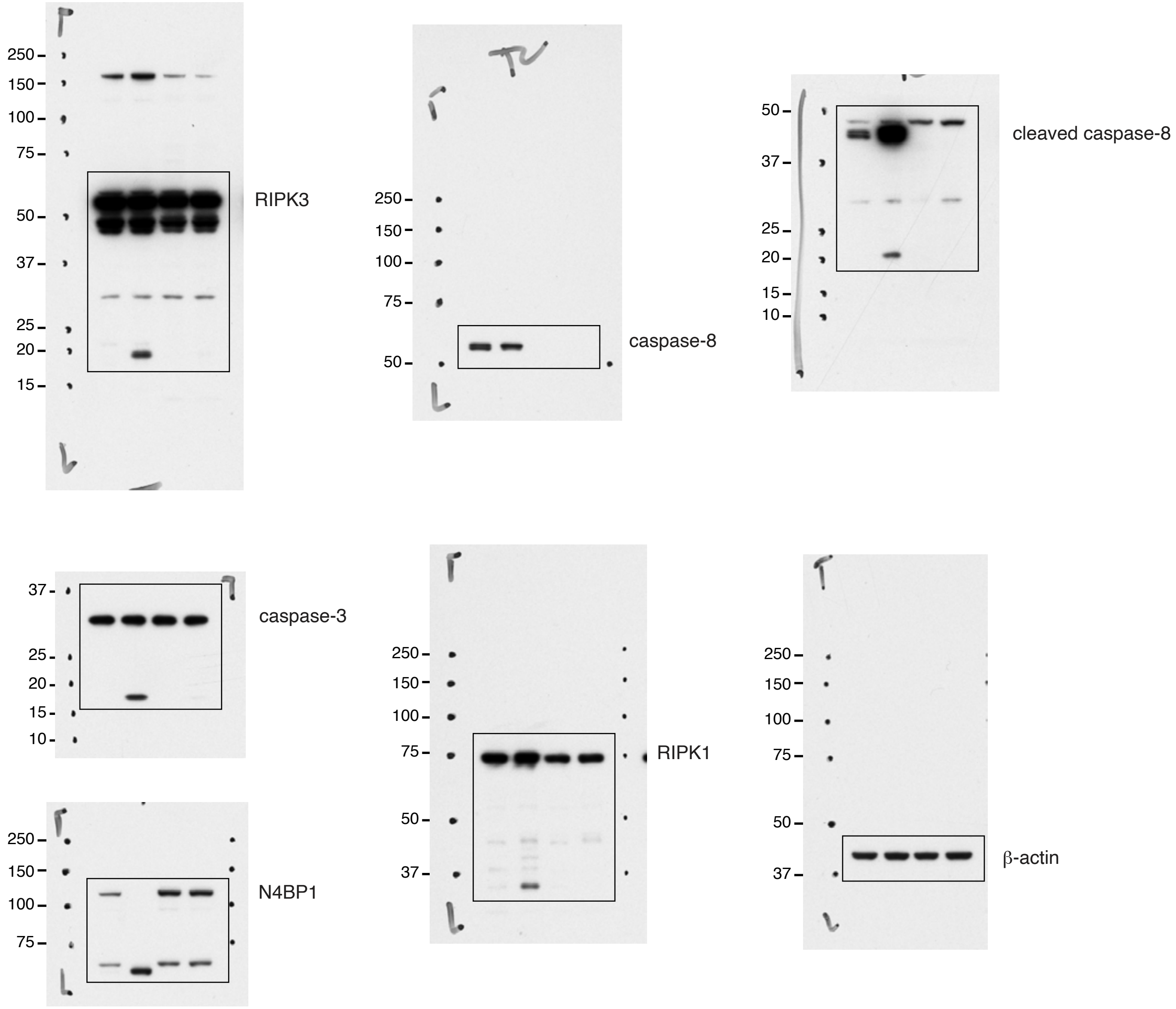

Figure S1c

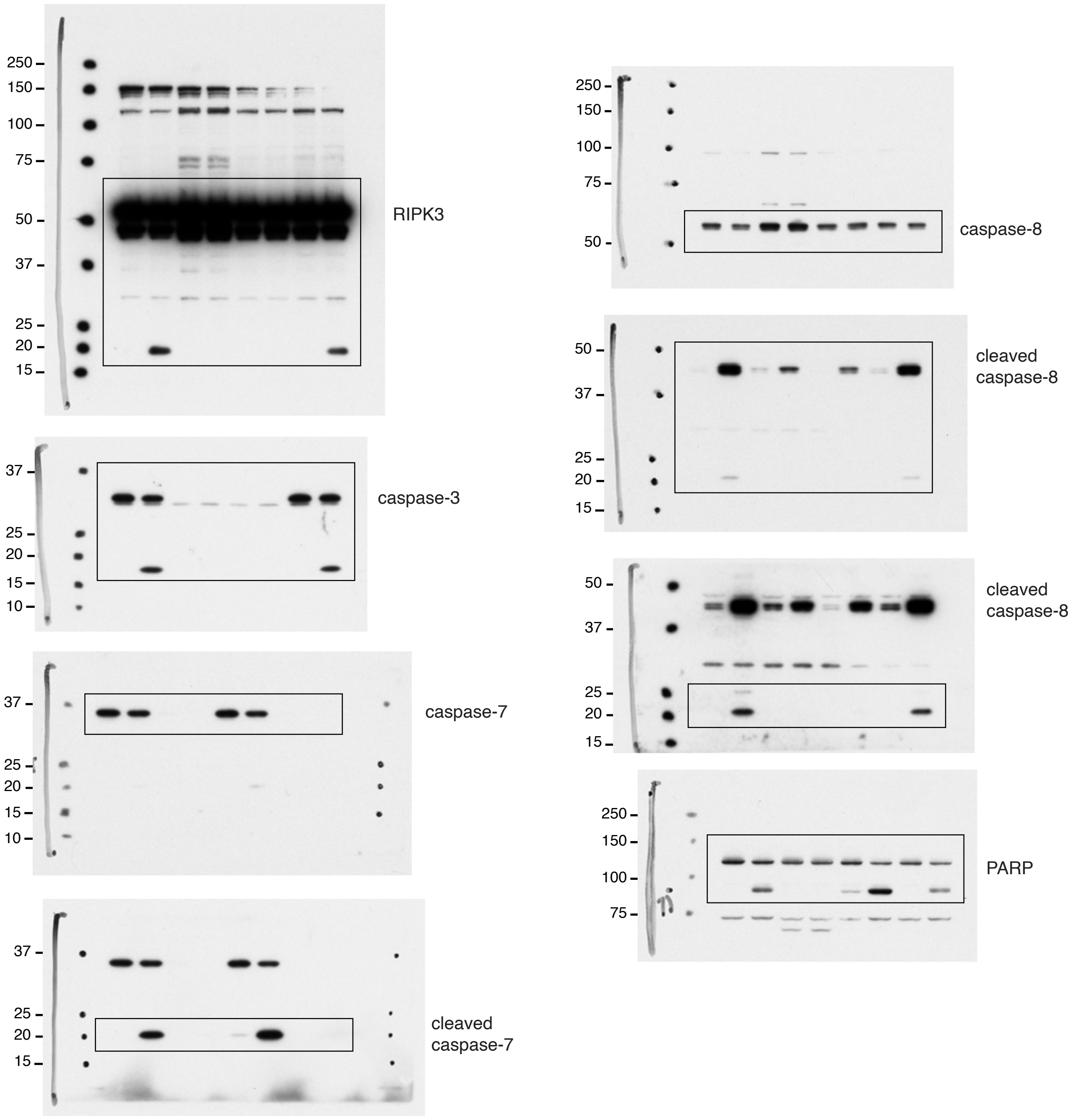
250
150
100
75
50
37
25
20
15
RIPK3
37
25
20
15
10
caspase-3
37
25
20
15
10
caspase-7
37
25
20
15
cleaved
caspase-7
250
150
100
75
50
caspase-8
50
37
25
20
15
cleaved
caspase-8
50
37
25
20
15
cleaved
caspase-8
250
150
100
75
PARP

Figure S1d

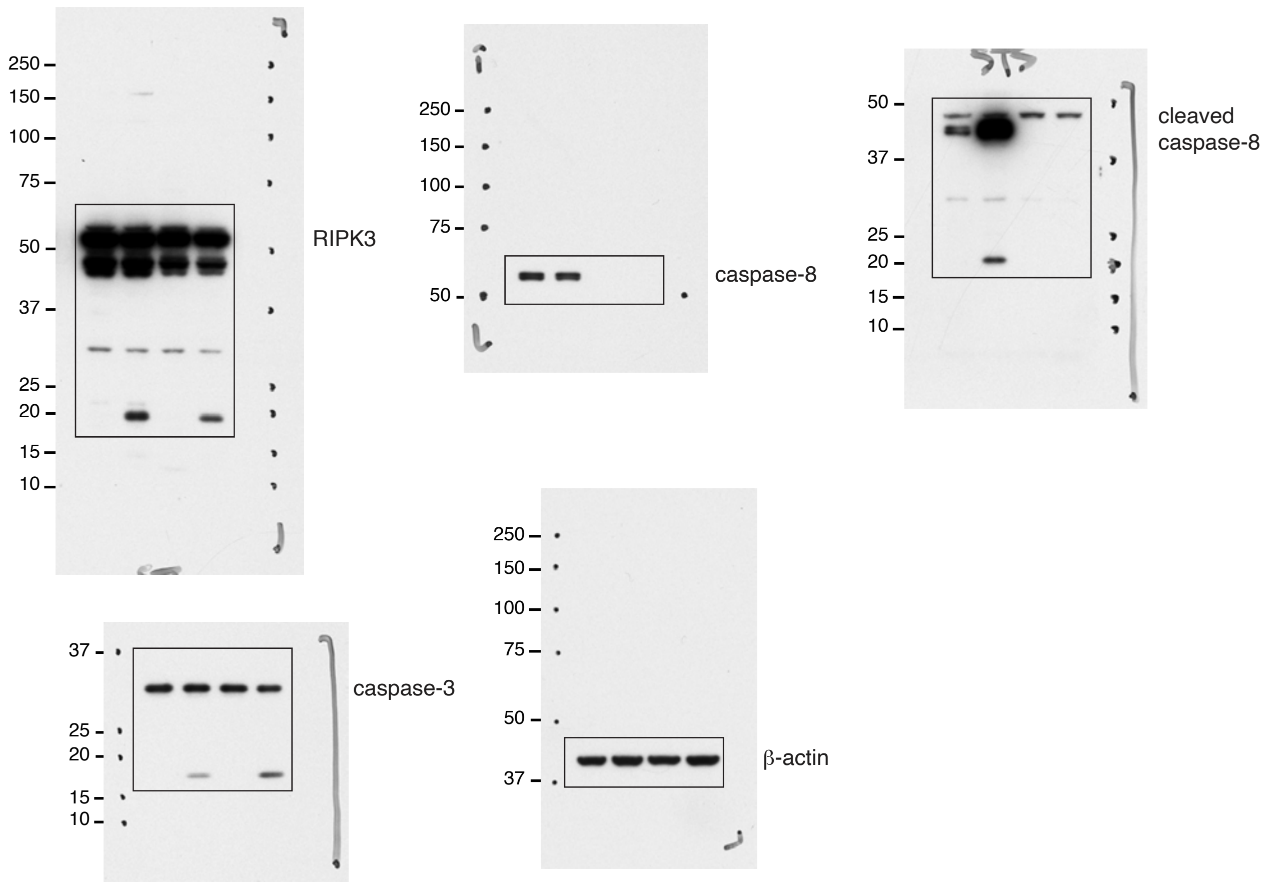

Figure S1e

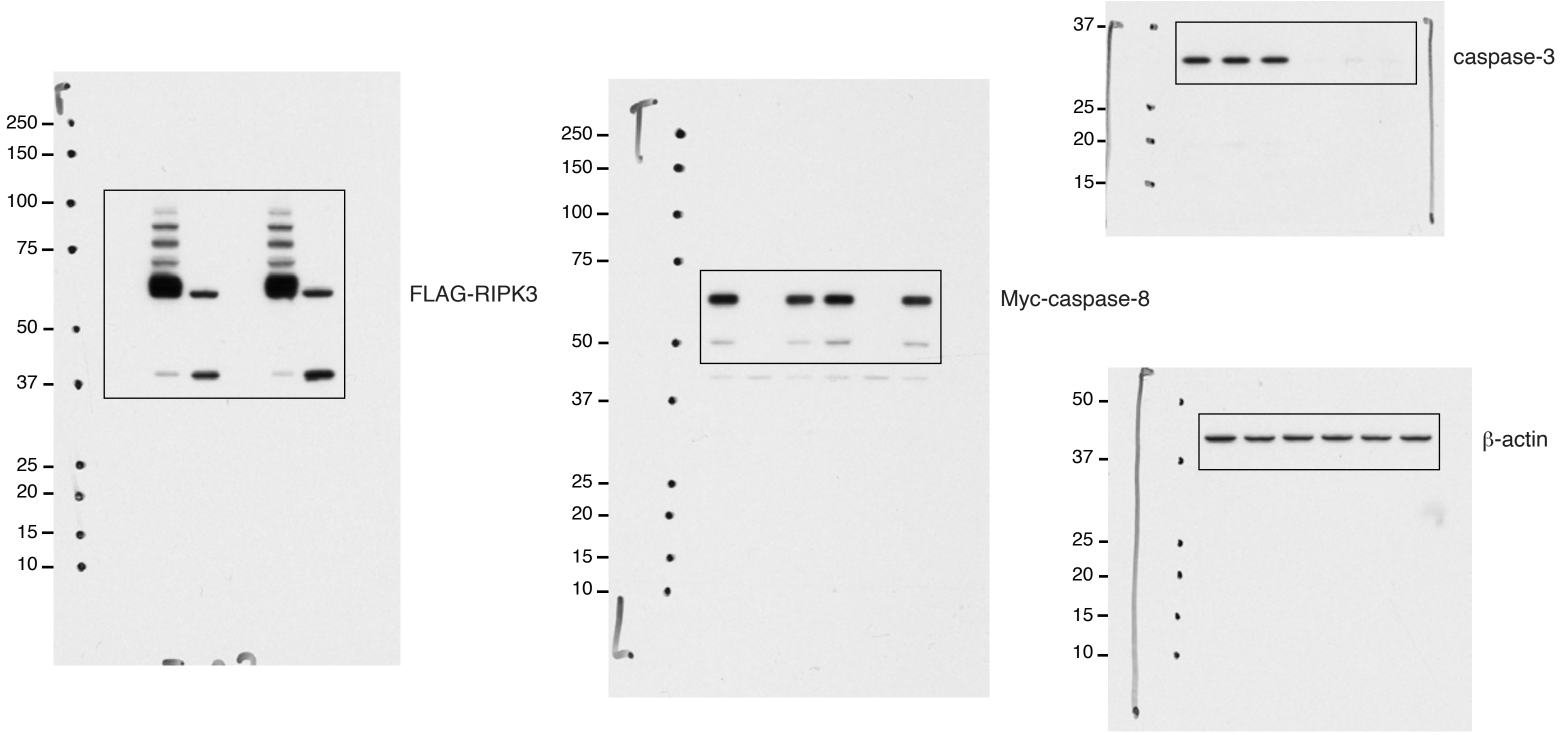

Figure S3a

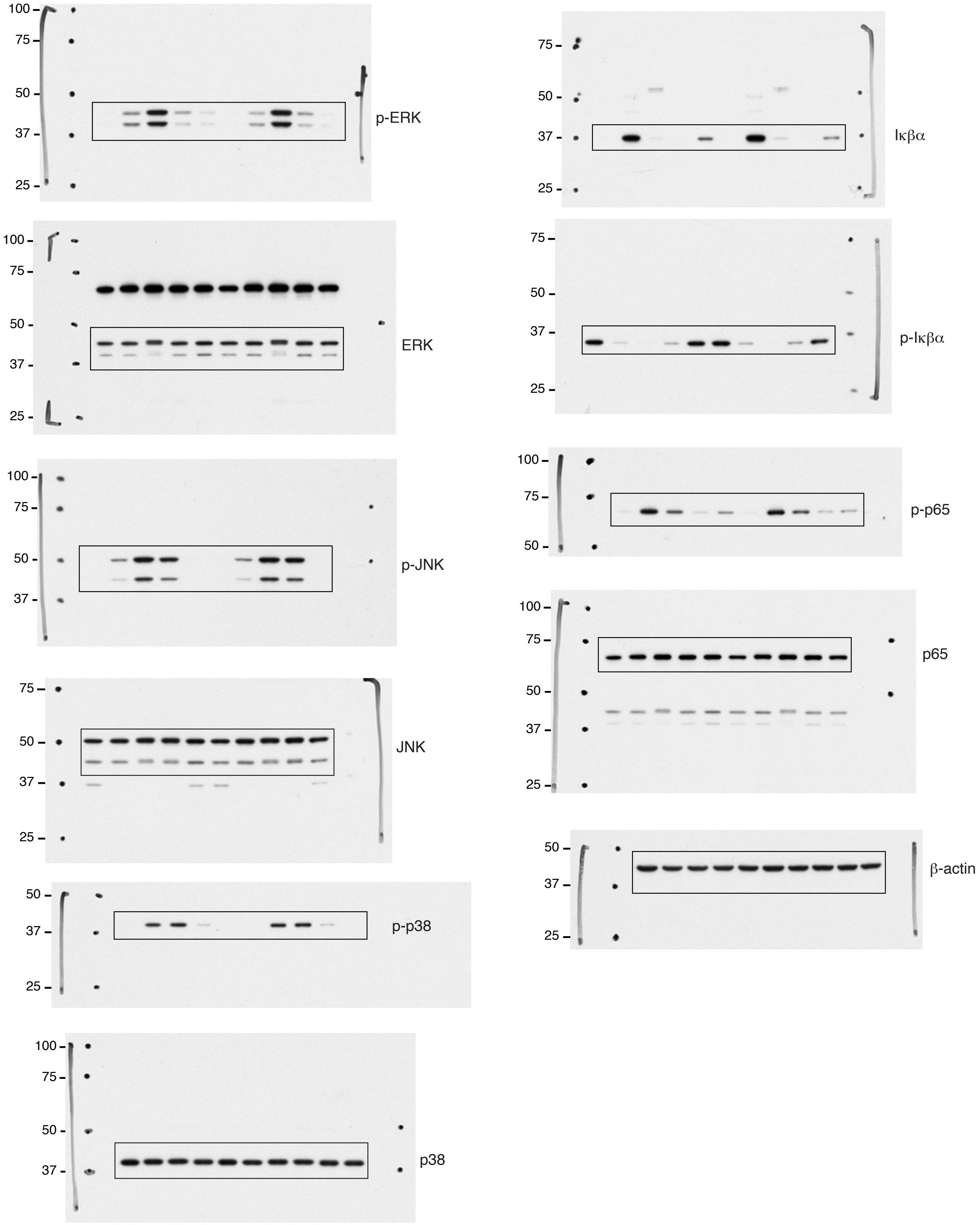
100
75
50
37
25
p-ERK
ERK
p-JNK
JNK
p-p38
p38
Iκβα
p-Iκβα
p-p65
p65
β-actin

Figure S3b

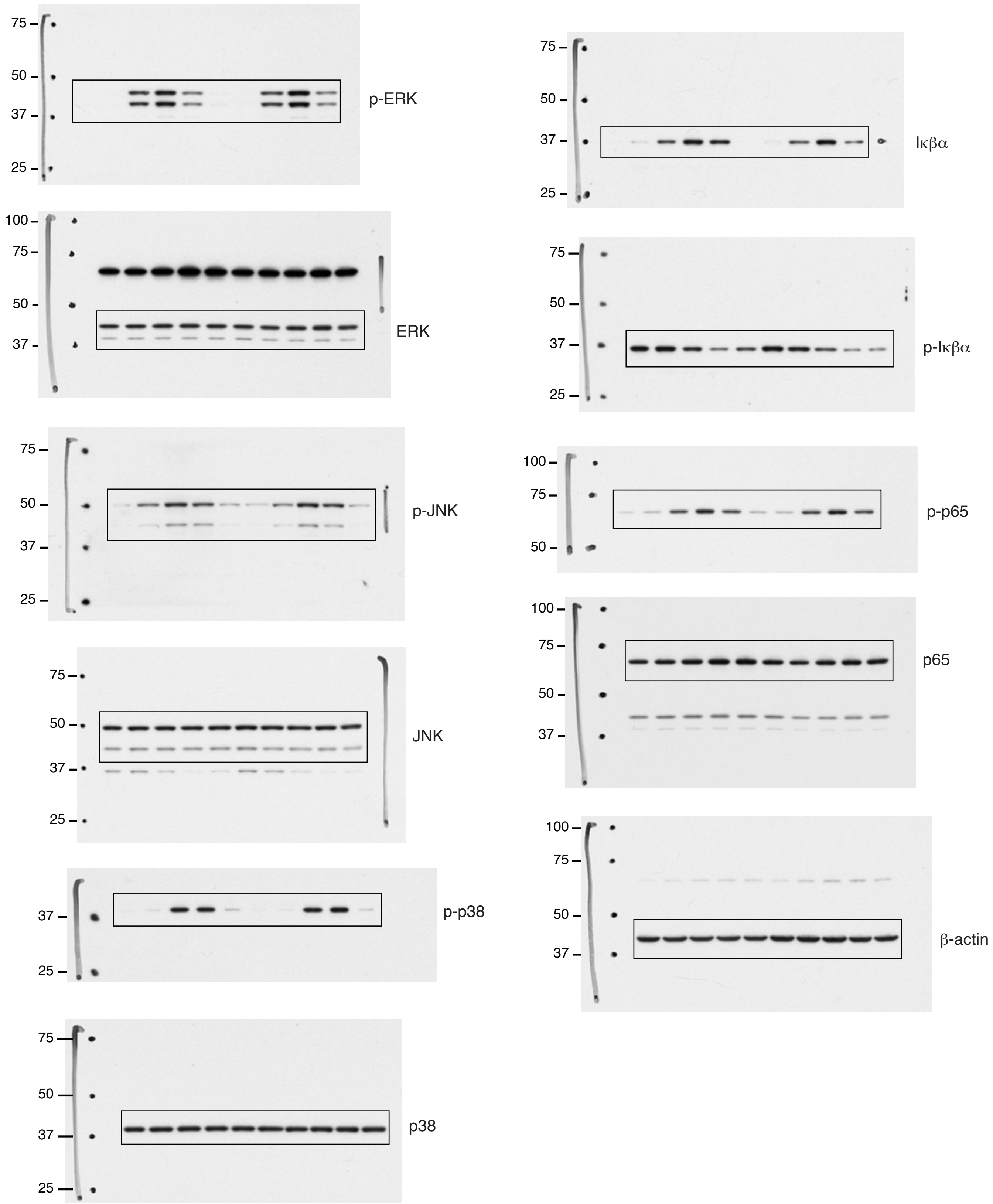
p-ERK
ERK
p-JNK
JNK
p-p38
p38
Iκβα
p-Iκβα
p-p65
p65
β-actin
100
75
50
37
25

Figure S3c

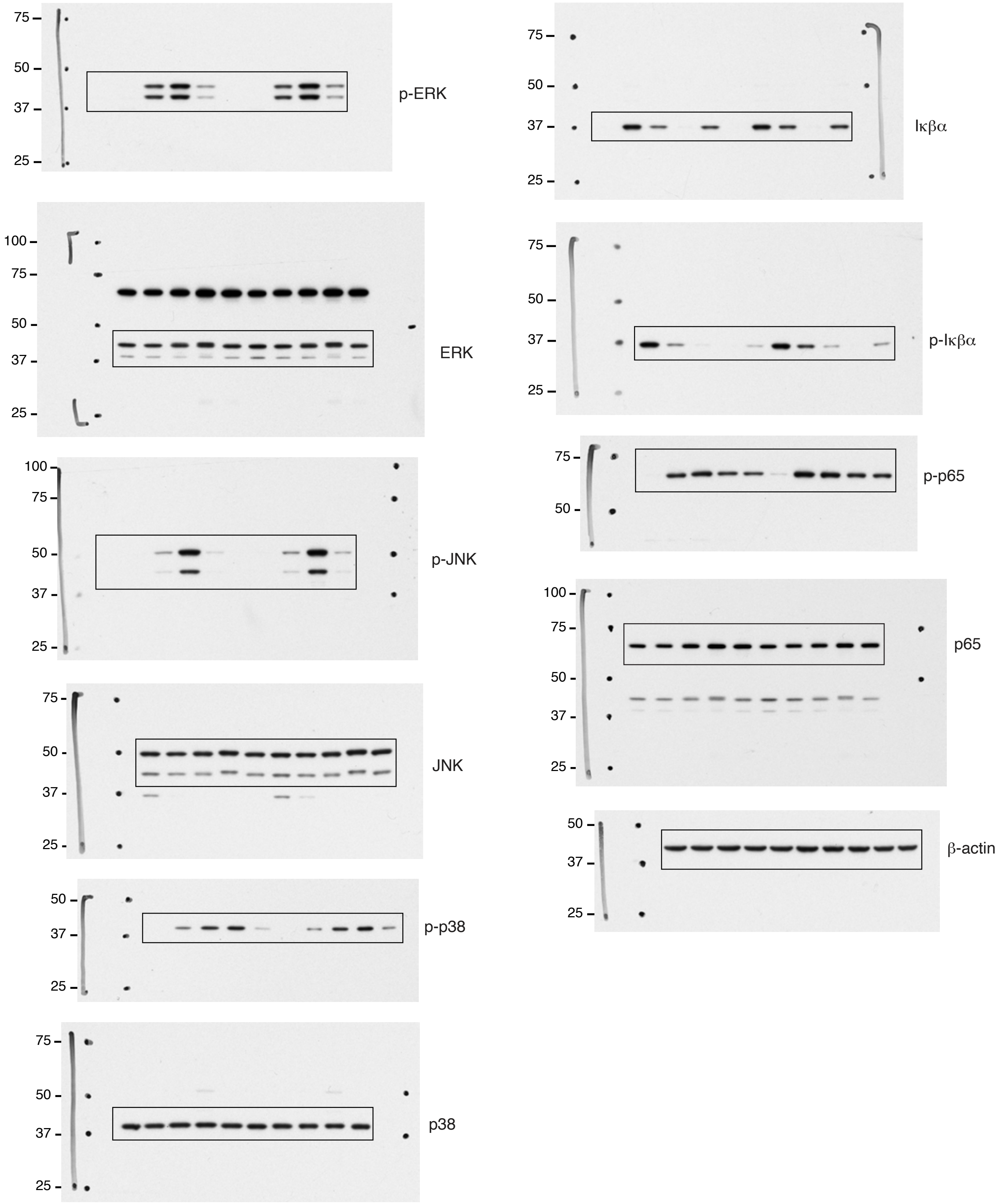
p-ERK
ERK
p-JNK
JNK
p-p38
p38
Iκβα
p-Iκβα
p-p65
p65
β-actin
100
75
50
37
25

Figure S4

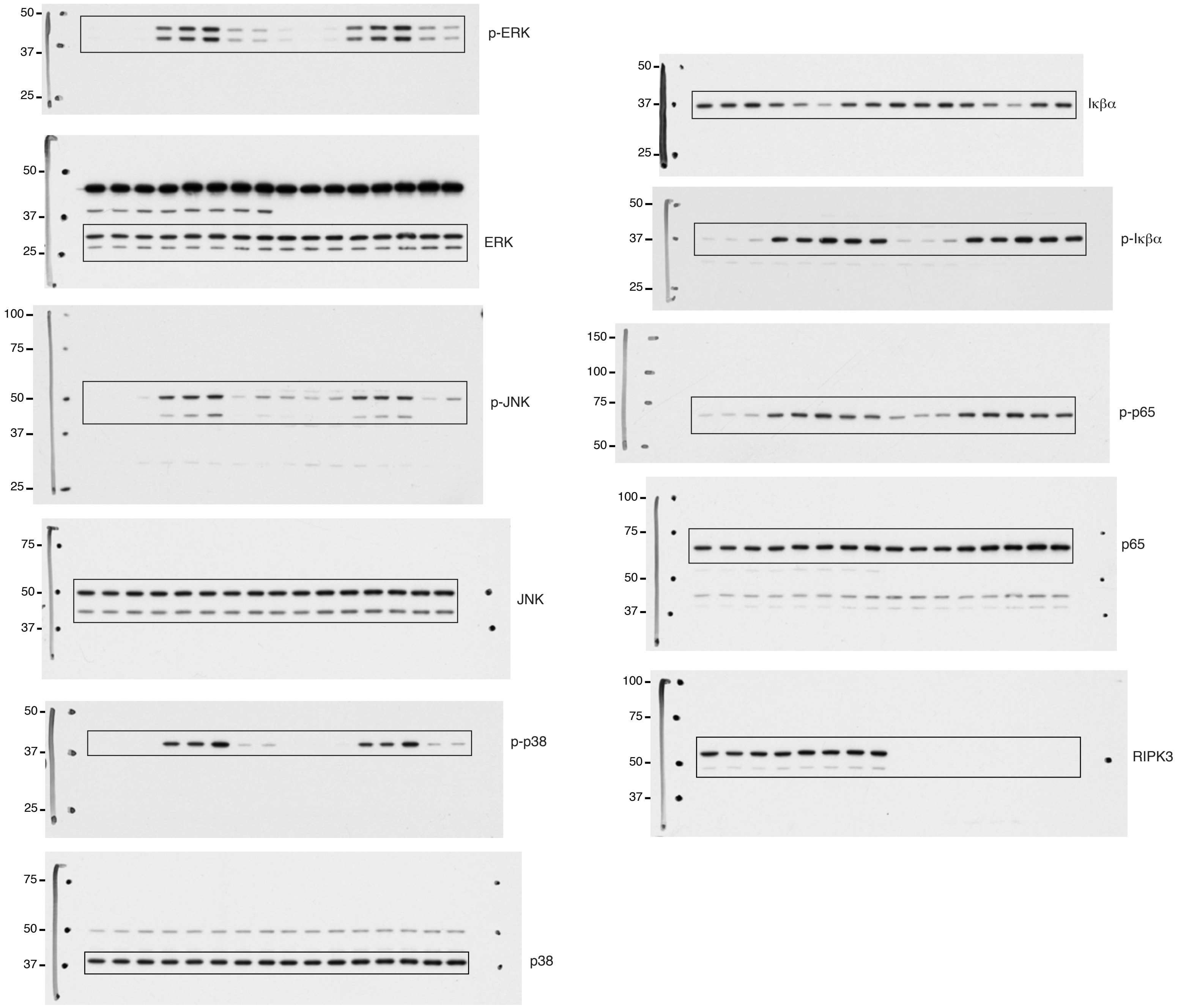

Supplement: Supplementary file 6 — Supplementary Figure 6 [file 41418_2023_1255_MOESM6_ESM.pdf]
